# Molecular Approach to Alkali-Metal Encapsulation by a Prussian Blue Analogue Fe^II^/Co^III^ Cube in Aqueous Solution: A Kineticomechanistic Exchange Study

**DOI:** 10.1021/acs.inorgchem.1c03001

**Published:** 2021-11-12

**Authors:** Miguel A. Gonzálvez, Paul V. Bernhardt, Mercè Font-Bardia, Albert Gallen, Jesús Jover, Montserrat Ferrer, Manuel Martínez

**Affiliations:** † School of Chemistry and Molecular Biosciences, University of Queensland, Brisbane, Queensland 4072, Australia; ‡ Secció de Química Inorgànica, Departament de Química Inorgànica i Orgànica, Universitat de Barcelona, Martí i Franquès 1−11, 08028 Barcelona, Spain; § Unitat de Difracció de Raigs, X. Centre Científic i Tecnològic,Departament de Cristal·lografia, and Mineralogia i Dipòsits Minerals, Facultat de Geologia, Universitat de Barcelona, 08028 Barcelona, Spain; ∥ Institut de Química Teòrica i Computacional, Universitat de Barcelona, 08028 Barcelona, Spain; ⊥ Institute of Nanoscience and Nanotechnology, Universitat de Barcelona, 08028 Barcelona, Spain

## Abstract

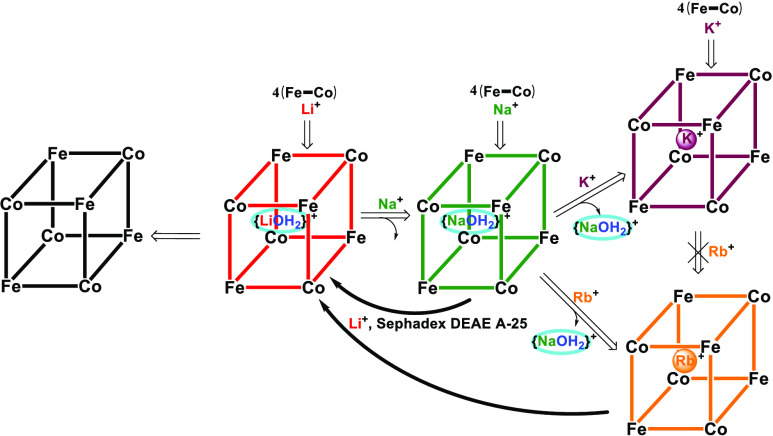

The preparation of a series of alkali-metal inclusion complexes of the molecular cube [{Co^III^(Me_3_-tacn)}_4_{Fe^II^(CN)_6_}_4_]^4–^ (Me_3_-tacn = 1,4,7-trimethyl-1,4,7-triazacyclononane), a mixed-valent Prussian Blue analogue bearing bridging cyanido ligands, has been achieved by following a redox-triggered self-assembly process. The molecular cubes are extremely robust and soluble in aqueous media ranging from 5 M [H^+^] to 2 M [OH^–^]. All the complexes have been characterized by the standard mass spectometry, UV–vis, inductively coupled plasma, multinuclear NMR spectroscopy, and electrochemistry. Furthermore, X-ray diffraction analysis of the sodium and lithium salts has also been achieved, and the inclusion of moieties of the form {M–OH_2_}^+^ (M = Li, Na) is confirmed. These inclusion complexes in aqueous solution are rather inert to cation exchange and are characterized by a significant decrease in acidity of the confined water molecule due to hydrogen bonding inside the cubic cage. Exchange of the encapsulated cationic {M–OH_2_}^+^ or M^+^ units by other alkali metals has also been studied from a kineticomechanistic perspective at different concentrations, temperatures, ionic strengths, and pressures. In all cases, the thermal and pressure activation parameters obtained agree with a process that is dominated by differences in hydration of the cations entering and exiting the cage, although the size of the portal enabling the exchange also plays a determinant role, thus not allowing the large Cs^+^ cation to enter. All the exchange substitutions studied follow a thermodynamic sequence that relates with the size and polarizing capability of the different alkali cations; even so, the process can be reversed, allowing the entry of {Li–OH_2_}^+^ units upon adsorption of the cube on an anion exchange resin and subsequent washing with a Li^+^ solution.

## Abstract 2

A series of alkali-metal inclusion complexes of the molecular cube [{Co^III^(Me_3_-tacn)}_4_{Fe^II^(CN)_6_}_4_]}]^4−^ have been achieved by following a redox-triggered self-assembly process. The alkali metals are encapsulated in an inert fashion as cationic {M−OH_2_}^+^ or M^+^ units, which can be exchanged in a process that has been studied kineticomechanistically.

## Introduction

Intensely colored transition-metal mixed-valence complexes such as Prussian Blue (or ferric ferrocyanide) have attracted the attention of both the chemical and general public communities for centuries. In fact, the general use of Prussian Blue as a resistant and fairly innocuous inorganic colorant cannot be overstated. As mixed-valence compounds, Prussian Blue analogues (PBAs) have been used academically for the establishment of fundamental mixed-valent classifications related to the electronic coupling between the metal centers and the symmetry-allowed inner-sphere optical electron transfer occurring between them.^1−8^ In reactivity aspects, these species have been utilized as cheap, metal-abundant catalysts in water oxidation processes^9,10^ and electrochemical applications.^11^ Recently, the use of these types of complexes in the development of photomagnetic switching materials has also been developed by several groups.^12−16^ Furthermore, both anionic Prussian Blue and PBAs are also known to act as hosts for cationic guests in general, which has led to various applications.^14,17−24^ Their use as sequestering agents has been explored, although their ion-exchange properties in aqueous solution are difficult to measure. Crown ether ligands offer a good perspective in this respect,^25^ but the lability of their complexes in aqueous solution hampers their “controlled” sequestering/bleaching reactions.^26^ The use and applicability of PBAs in medicinal chemistry have also been recently reviewed.^27^

The boundaries between discrete molecular, polymeric, and solid-state PBAs are rather diffuse.^12,28−30^ In this respect, solubility plays also a crucial role in the development and use of these types of compounds, especially when water is the solvent of choice, as recommended in Green Chemistry applications. In general, these compounds tend to become less soluble as their nuclearity increases. Furthermore, the solubility/stability issues of discrete molecular PBAs tend to require aprotic and noncoordinating solvents to avoid destruction of the 2D or 3D structures due to the substitution lability of the metal centers.^30,31^ The possible use of PBAs as electron reservoirs/sinks is also an important point to be considered for their applications.^10,23,32^ For these purposes, the compounds require substitution inertness to avoid dissociation and loss of the components of this functional assembly.

**Chart 1:**
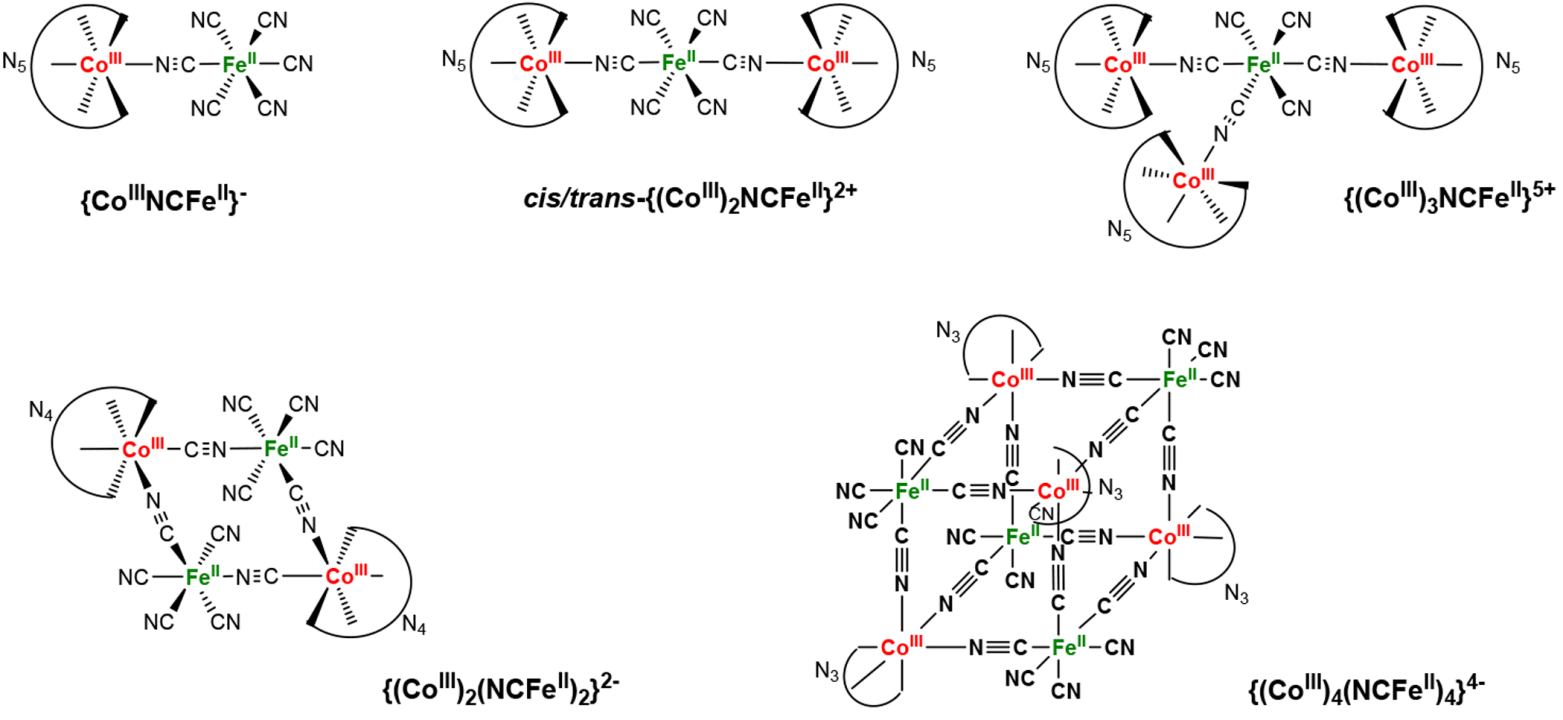


Synthesis of PBAs in a controlled and reproducible manner is vital for any future application. Many members of this family are isolated as crystalline but insoluble polymeric compounds (metal–organic frameworks) with well-defined structures based on X-ray diffraction (XRD) data, but establishment of the precise and rational preparative conditions is challenging. Alternative preparative procedures involving designed self-assembly aim to avoid such difficulties. The validity of the approach has been proven on a wealth of occasions for several families of compounds.^33,34^ We have been involved for some time in the study of the self-assembly and reactivity of 3D oligonuclear structures, including the study of their formation and dynamic behavior.^35−38^ A concerted redox-triggered ligand substitution approach has led to the family of complexes indicated in Chart 1, where cyanido ligands bridge the metal centers and the remaining coordination sites are occupied by a macrocyclic tri-, tetra-, or pentaamine (N_3_, N_4_, or N_5_).^39−45^ These cobalt/iron PBAs behave as discrete molecular Class II mixed-valence complexes with remarkable thermal and pH stability, as well as reversible redox reactivity;^44,46−49^ this chemistry has successfully included other metals as well.^40,50−52^ Potential applications of the redox properties of these complexes on inert supports have also been explored.^53,54^

In this report, we focus on the inclusion properties of the recently communicated molecular cube in Chart 1 (N_3_ = 1,4,7-trimethyl-1,4,7-triazacyclononane, Me_3_-tacn).^45^ The alkali-metal and water exchange properties have been studied by time-resolved UV–vis spectroscopy and multinuclear NMR measurements at variable concentrations, ionic strengths, temperatures, and pressures in order to obtain the activation parameters for the process. These proved to be dominated by solvation/desolvation of the different cations upon exiting/entering the cubic cage. This is a fact that had not been explored precisely previously because of a lack of the desirable aqueous chemistry of these molecular PBAs. Furthermore, we observed a particularly unexpected reactivity on chromatography-adsorbed samples, which was not paralleled in solution. This reveals an interesting heterogeneous effect on the encapsulation reactivity of the highly negatively charged cubic structure. Density functional theory (DFT) calculations have been employed to assess the structural features of the cubic complexes with encapsulated alkaline cations containing different numbers of water molecules.

## Results

### Preparation of Compounds

The lithium, sodium, and potassium salts of the [{Co^III^(Me_3_-tacn)}_4_{Fe^II^(CN)_6_}_4_]^4–^ mixed-valence molecular cubes have been prepared using the well-developed mechanistically directed self-assembly process described in previous literature reports for 2D structures (Chart 1).^39,41−44,49,51,55^ A preliminary communication on the preparation of sodium and potassium salts of the cube was also reported.^45^ In all cases, preparations have been conducted, as indicated in the Experimental Section, by the reaction of [Co^III^(Me_3_-tacn)Cl_3_], with the desired [Fe^II^(CN)_6_]^4–^ salt in aqueous solution at 50 °C overnight. From this point, the solutions were treated as indicated below to obtain the various salts of the mixed-valence cube.

### Lithium Salt

**Figure 1:**
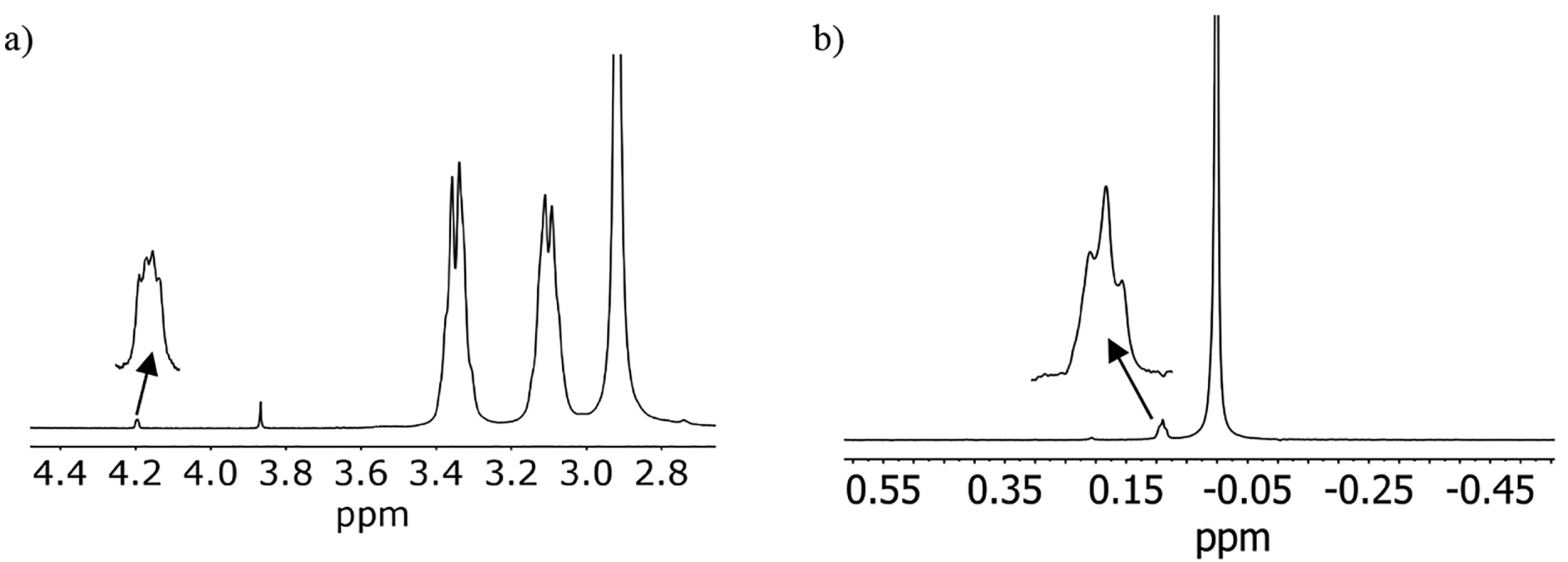
(a) ^1^H NMR spectrum of the Sephadex G-25 eluate obtained from the crude mixture of the preparation of the lithium salt of the [{Co^III^(Me_3_-tacn)}_4_{Fe^II^(CN)_6_}_4_]^4–^ cubic cage. (b) ^7^Li NMR spectrum of the same sample.

**Figure 2:**
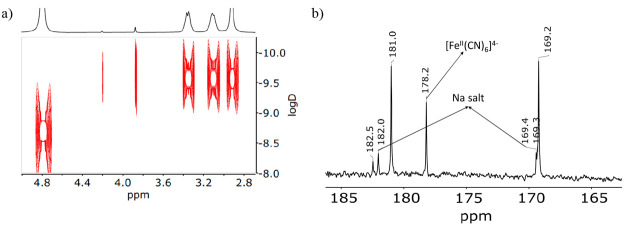
(a) DOSY NMR spectrum of the sample obtained from the crude mixture of the preparation of the lithium salt of the [{Co^III^(Me_3_-tacn)}_4_{Fe^II^(CN)_6_}_4_]^4–^ structure. (b) ^13^C NMR spectrum of the same sample.

The procedure for obtention of the lithium salt of [{Co^III^(Me_3_-tacn)}_4_{Fe^II^(CN)_6_}_4_]^4–^ parallels that used for the reported preparation of its sodium or potassium salts^45^ but using (see the experimental part) Li_4_[Fe^II^(CN)_6_] as the source of the {Fe^II^(CN)_6_} building block. An initial Sephadex G-25 chromatographic workup, to eliminate any excess cobalt or iron building blocks, produced a purple solution that, after being taken to dryness at 35–45 °C, exhibits a ^1^H NMR spectrum with signals of the symmetric {Co^III^(Me_3_-tacn)} moiety *plus* significant peaks of low intensity (with respect to those of the Me_3_-tacn moiety) at 4.21 and 3.87 ppm (Figure 1a).^56^ The signal at 3.87 ppm is associated with water bound to residual amounts of Na^+^ in the building block samples, which led to formation of the already described sodium salt of the cubic structure.^45^ As for the signal at 4.21 ppm, it shows a very distinct line shape that is associated with coupling to a *I* = ^3^/_2_ spin nucleus in a very symmetrical environment, as expected for a small ^7^Li center (*J*_H–Li_ = 1.1 Hz). Furthermore, the chemical shift (4.21 ppm), compared with that of encapsulated {Na–OH_2_}^+^ (3.87 ppm), is consistent with a more acidic water molecule, as expected from the higher polarizing power of the attached lithium cation. In this respect, the ^7^Li NMR spectrum of the same sample shows a signal at 0 ppm, from aquated lithium cations, *plus* a triplet at 0.09 ppm (*J*_Li–H_ = 1.1 Hz; Figure 1b) indicative of the presence of an encapsulated {Li–OH_2_}^+^ unit. Figure 2a shows the DOSY NMR spectrum of the same sample, which corroborates the inclusion of either the {Li–OH_2_}^+^ (4.21 ppm) or {Na–OH_2_}^+^ (3.87 ppm) units within their corresponding cages. The ^13^C NMR spectrum of the same sample is even more revealing, showing the presence of three sets of resonances in the cyanide region corresponding to cubic structures, *plus* that of the remaining [Fe^II^(CN)_6_]^4–^, still present in the crude mixture obtained under the experimental conditions used (Figure 2b).

The ^1^H and ^7^Li NMR experiments indicate that the minor component (the one having the least intense signals) corresponds to a structure with encapsulated {Li–OH_2_}^+^ units, while the major component (that with the most intense signals) has no encapsulated lithium and/or water units, i.e., void. Interestingly, the ^1^H NMR spectrum of an aged solution (6–8 days, room temperature) of this sample shows practically no signal associated with the species containing encapsulated {Li–OH_2_}^+^ units, thus indicating that the void structure is formed upon prolonged standing.^57^ No further attempts to obtain a pure sample of an encapsulating {Li–OH_2_}^+^ cube were pursued under these conditions, although an enriched and inert, lithium-containing cube was accomplished by cation-exchange chromatography, as indicated in the following section.

**Figure 3:**
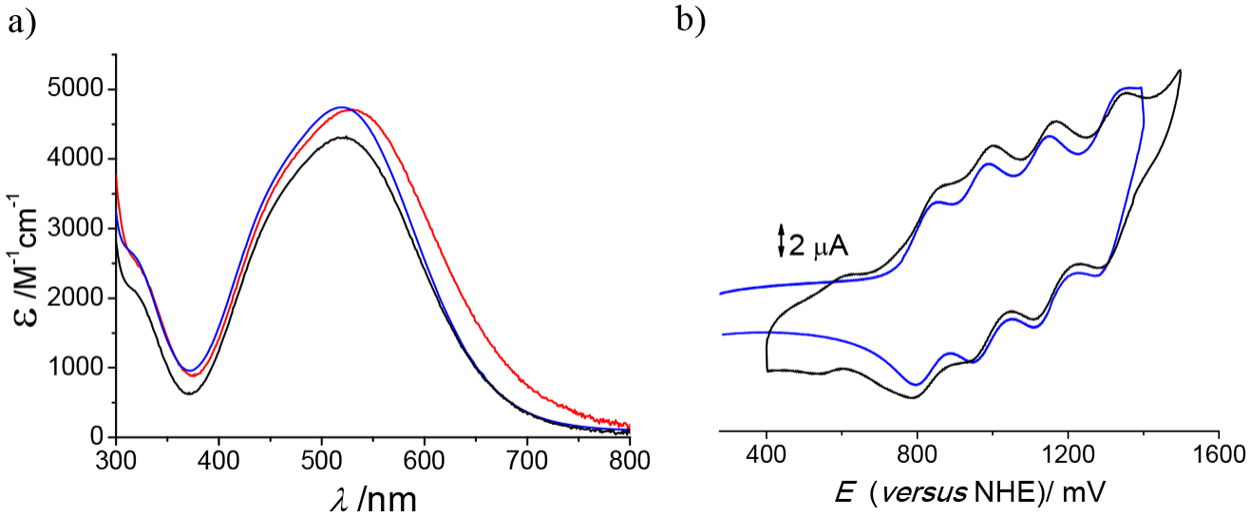
(a) Electronic spectra of the major component (i.e., void) of the lithium (black), sodium (red), and potassium (blue) salts of the [{Co^III^(Me_3_-tacn)}_4_{Fe^II^(CN)_6_}_4_]^4–^ species in water. (b) Cyclic voltammograms of the Fe^III^/Fe^II^ region of the major component (i.e., void) of the lithium salt (black; the minor signal at ca. 500 mV is associated with the residual sodium salt of the species) and potassium salt (blue) of the [{Co^III^(Me_3_-tacn)}_4_{Fe^II^(CN)_6_}_4_]^4–^ cubic cage.

The major component of the mixture was isolated by further Sephadex G-25 size-exclusion column chromatography, discarding the head and tail of the band. The ^7^Li NMR spectrum of this eluate shows only the signal of solvated lithium cations, thus indicating that the major component of the mixture effectively corresponds to the void architecture; its ^13^C NMR spectrum also indicates the distinct uniqueness of the species (Figure S1a). In this respect, the lithium-to-sodium exchange of this fraction, conducted by Sephadex DEAE A-25 anion-exchange chromatography, produced a sample with no lithium ions (as shown by inductively coupled plasma (ICP) analysis). ICP metal analysis of this compound allowed a comparison of the UV–vis spectra of the prepared structures (Figure 3a). The sample of the major component (i.e., void) thus obtained was characterized by cyclic voltammetry (CV) experiments (Figure 3b). Four consecutive reversible Fe^III^/Fe^II^ responses are apparent in the {Fe^II^(CN)_6_} oxidation zone, indicating that the structure is rather similar to that reported for the potassium salt (no encapsulated water). CV shows some signals (see below and ref (45)) from the presence of residual sodium ions in the building block materials (as seen in the ^1^H NMR spectrum in Figure S1b).

**Figure 4:**
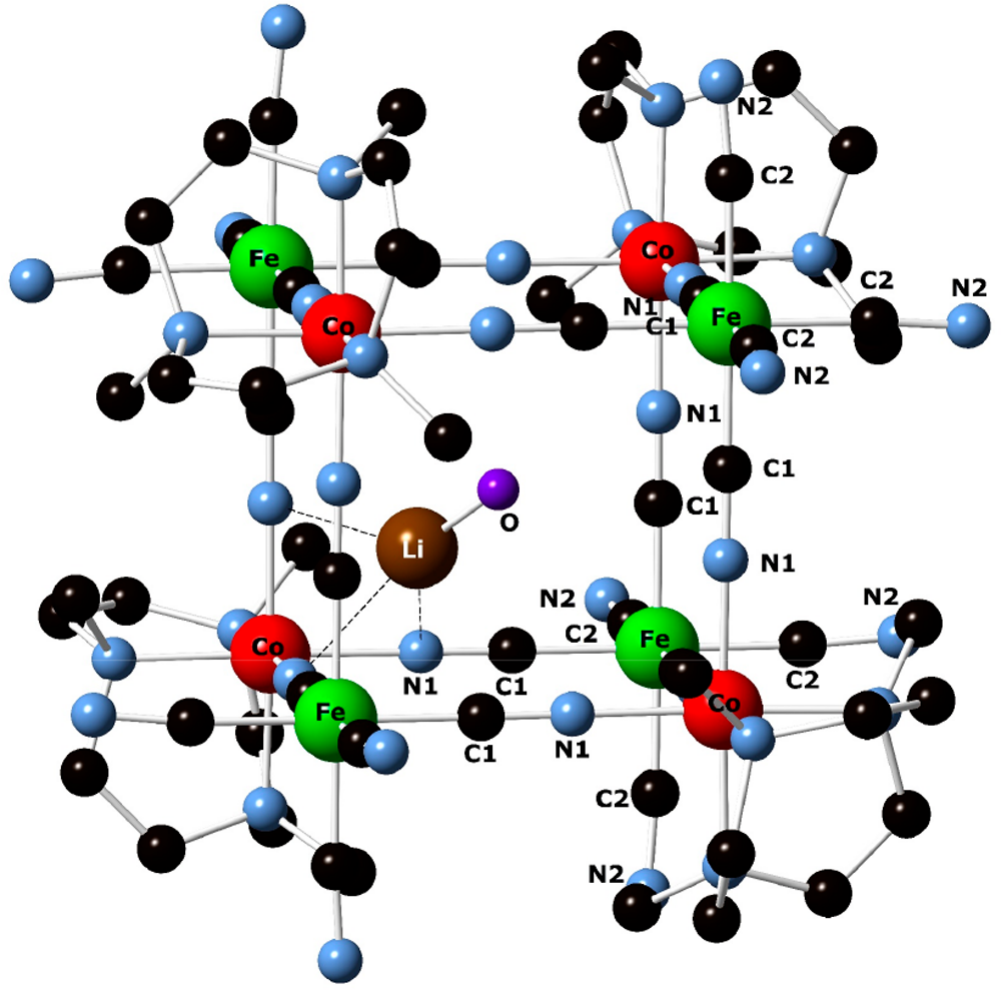
Ball-and-stick representation of the cubic {{Li–OH_2_}⊂[{Co^III^(Me_3_-tacn)}_4_{Fe^II^(CN)_6_}_4_]} structure of the Li_8_{{Li–OH_2_}⊂[{Co^III^(Me_3_-tacn)}_4_{Fe^II^(CN)_6_}_4_]}·(ClO_4_)_5_·12H_2_O compound showing the encapsulated {Li–OH_2_}.

In summary, the major kinetic product obtained using Li_4_[Fe^II^(CN)_6_] as {Fe^II^(CN)_6_} building block has encapsulated {Li–OH_2_}^+^ units that leach from the cube into the bulk solution upon workup and long-standing, producing a thermodynamically stable void cubic {{Co^III^(Me_3_-tacn)}_4_{Fe^II^(CN)_6_}_4_} structure. Even so, the enrichment of a sample of {{Co^III^(Me_3_-tacn)}_4_{Fe^II^(CN)_6_}_4_} containing {Li–OH_2_}^+^ units was achieved by cation exchange on a Sephadex DEAE A-25 resin (see the Adsorption Cation-Exchange Study section), and full characterization of {{Li–OH_2_}⊂[{Co^III^(Me_3_-tacn)}_4_{Fe^II^(CN)_6_}_4_]} was thus accomplished. Figure 4 displays the X-ray crystal structure of the cube Li_8_{{Li–OH_2_}⊂[{Co^III^(Me_3_-tacn)}_4_{Fe^II^(CN)_6_}_4_]}·(ClO_4_)_5_·12H_2_O compound, which is in agreement with the spectroscopic characterization data available.

A very similar structure was determined for this complex via DFT calculations. The results from these produce a compound where the lithium atom interacts with three nitrogen atoms of the cyanido bridging groups (average Li–N distance = 2.18 Å) and the water molecule occupies the center of the cavity. This water molecule also shows four hydrogen-bonding interactions with different cyanido bridging ligands (average H···N distance = 2.55 Å). Interestingly, other species containing a lithium cation *plus* two or no water molecules were also computed, producing compounds with higher Gibbs energies (13.6 and 25.4 kcal mol^–1^, respectively), in excellent agreement with the experimental characterization of the complex encapsulating a {Li–OH_2_}^+^ unit. The hypothetical complex containing a {Li_2_OH_2_}^2+^ unit was also computed but found to be 10.2 kcal mol^–1^ higher in Gibbs energy.

**Figure 5:**
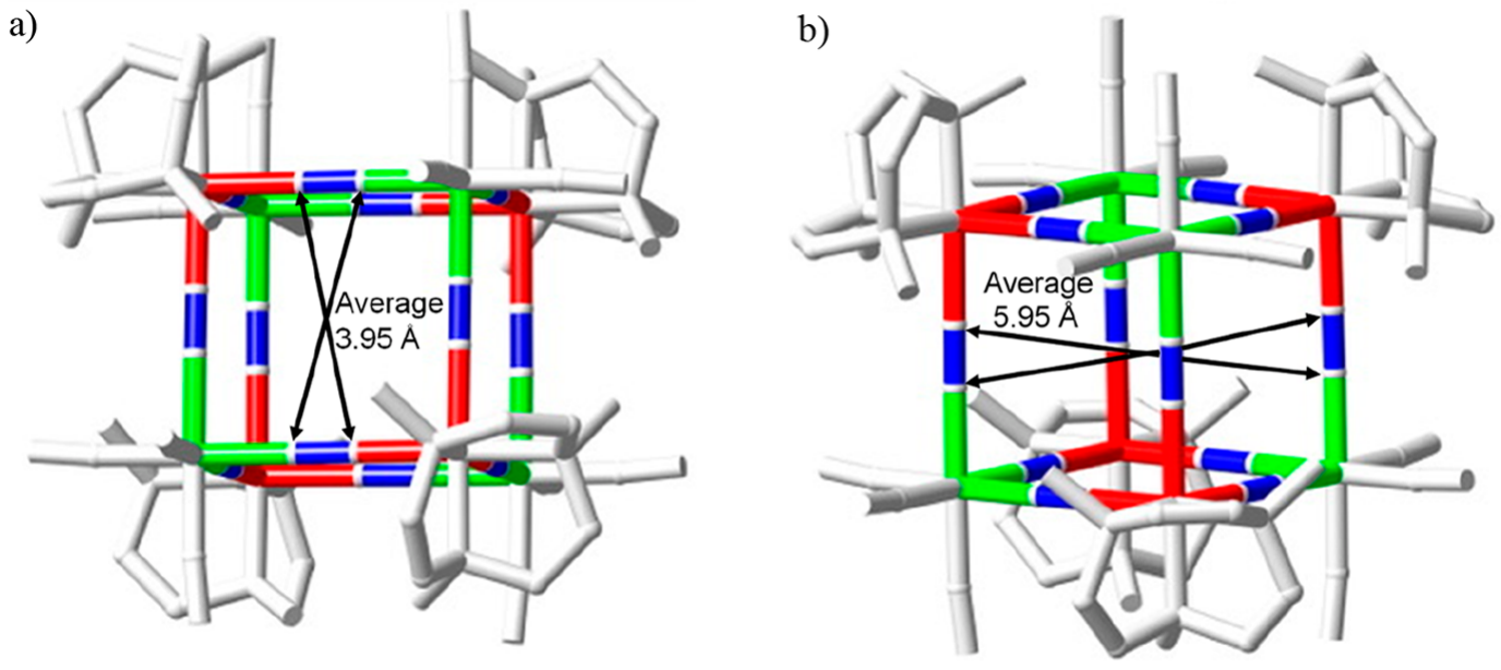
Relevant portal (a) and cavity (b) dimensions of the cubic cage of the Li_8_{{LiOH_2_}⊂[{Co^III^(Me_3_-tacn)}_4_{Fe^II^(CN)_6_}_4_]}·(ClO_4_)_5_·12H_2_O compound.

Figure 5 shows the relevant maximum portal and cavity sizes of the cube, where the radii of the carbon (0.60 Å) and nitrogen (0.54 Å) atoms of the cyanido groups have been taken from the literature data.^58^ The values indicate that a sphere of a maximum radius of ∼2 Å could be simplistically made to go through the square portal of the cube and one of ∼3 Å would fit inside the cubic cage.

**Figure 6:**
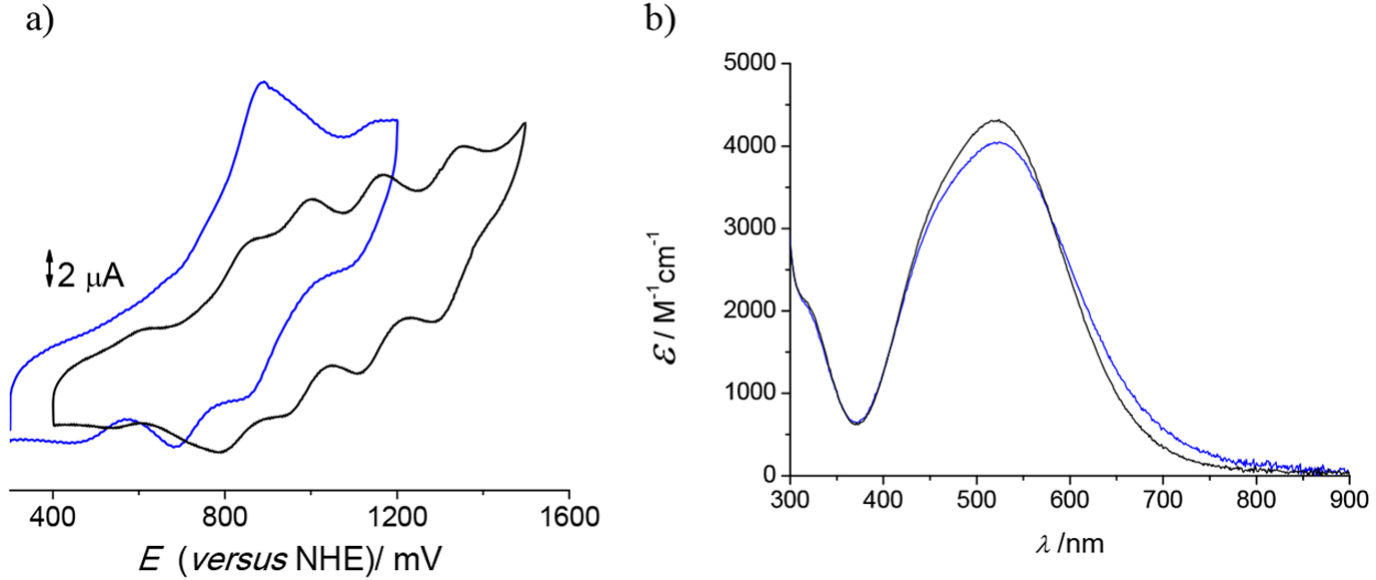
(a) Cyclic voltammograms of the Fe^III^/Fe^II^ signals of the void (black) and {Li–OH_2_}^+^-containing (blue) lithium salts of the [{Co^III^(Me_3_-tacn)}_4_{Fe^II^(CN)_6_}_4_]^4–^ cubic cages. (b) Electronic spectra of the same samples.

Figure 6 collects the comparison between the UV–vis spectrum and cyclic voltammogram of the void and {Li–OH_2_}^+^-containing compounds prepared, showing clear differences between samples with and without inert water confined. It is interesting to note that, upon repetitive scanning, the cyclic voltammogram of the {Li–OH_2_}^+^-containing lithium salt cage evolves to that of the void cube (Figure 3a), thus indicating that oxidation of the structure effectively labilizes the {Li–OH_2_}^+^ units.

### Sodium Salt

The general procedure for the preparation of the sodium salt of the [{Co^III^(Me_3_-tacn)}_4_{Fe^II^(CN)_6_}_4_]^4–^ complex, as well as its characterization by ^1^H and ^13^C NMR, UV–vis spectroscopy, IR spectrometry, inductively coupled plasma optical emission spectrometry (ICP-OES), electrospray ionization mass spectrometry (ESI-MS), and electrochemistry, has already been described.^45^

**Figure 7:**
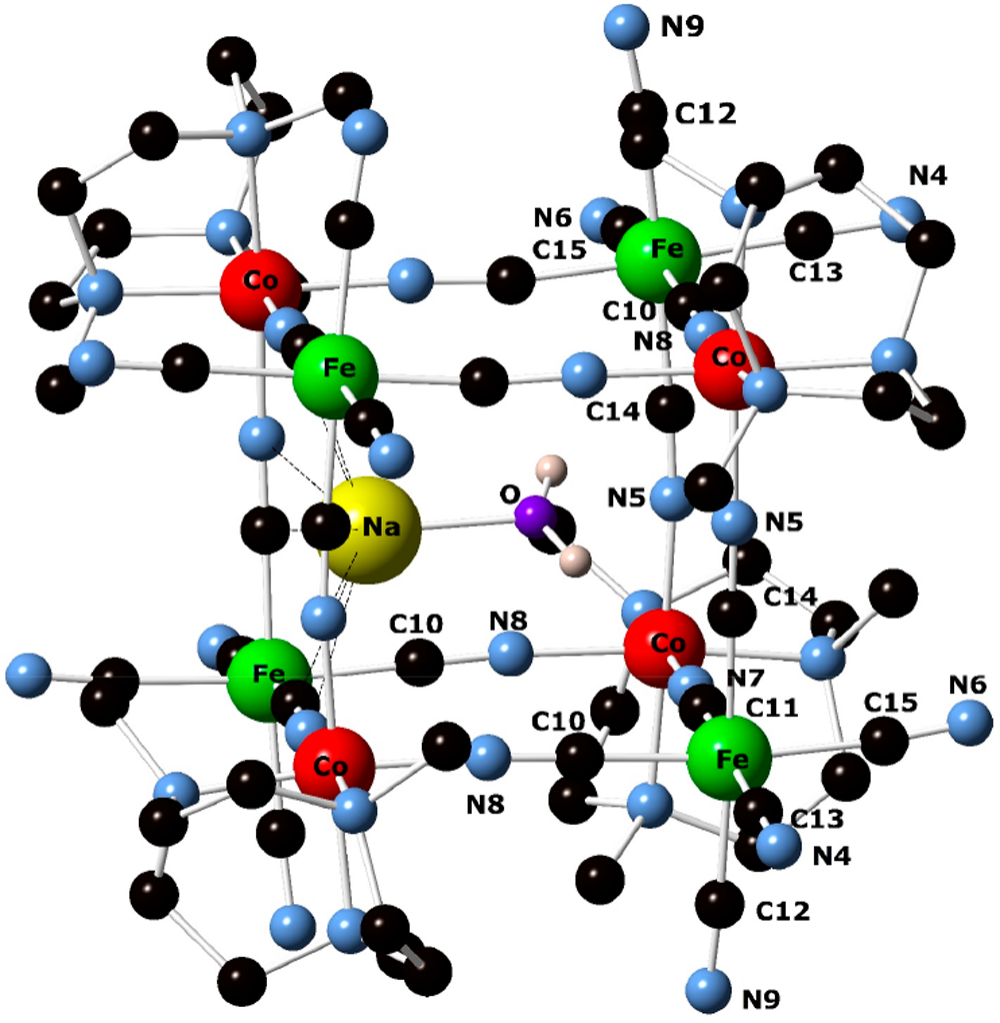
Ball-and-stick representation of the cubic {{Na–OH_2_}⊂[{Co^III^(Me_3_-tacn)}_4_{Fe^II^(CN)_6_}_4_} structure of the Na_3_{{Na–OH_2_}⊂[{Co^III^(Me_3_-tacn)}_4_{Fe^II^(CN)_6_}_4_]}·22H_2_O compound showing the encapsulated {Na–OH_2_}.

Because the solid compound always contains variable amounts of sodium perchlorate (arising from the anionic column chromatography workup), a purification procedure was conducted, taking advantage of the robustness of the compound at diverse pH values. A dissolved sample of the compound was treated with concentrated HCl up to 5 M, which led to precipitation of the protonated neutral compound,^44,45^ and the off-brown solid obtained was dissolved in 0.05 M NaOH after centrifugation. Slow evaporation of the solution in air, which slowly concentrates to pH 13–14, produced crystals of XRD quality that were subsequently analyzed as Na_3_{{Na–OH_2_}⊂[{Co^III^(Me_3_-tacn)}_4_{Fe^II^(CN)_6_}_4_]}·22H_2_O. Figure 7 displays the cubic {{Co^III^(Me_3_-tacn)}_4_{Fe^II^(CN)_6_}_4_} structure with the encapsulated {Na–OH_2_}^+^ unit, which is disordered within the cube. Although the quality of the crystals obtained, their high symmetry, and the large number of atoms and disordered water molecules involved do not allow for an ideal XRD analysis of the full structure, the confined {Na–OH_2_}^+^ unit is well-resolved, which validates the relevant chemistry involved. In contrast to the {Li–OH_2_}^+^ analogue (Figure 4), where the cation occupies one corner of the cube contacting three nitrogen atoms of bridging CN^–^ ligands and adopts a distorted tetrahedral geometry, the larger Na^+^ ion on the face of the cube makes four weak contacts with the cyanide nitrogen atoms to adopt a square-pyramidal coordination geometry with its axially coordinated aqua ligand.

This structure has also been proven with DFT calculations. In the calculated compound, the sodium atom is close to one of the inner faces of the cubic structure, interacting with the nitrogen atoms of four bridging CN^–^ ligands (average distance = 2.70 Å). The water molecule occupies the center of the cavity and interacts with the nitrogen atom of two CN^–^ groups (average distance = 2.24 Å). The DFT calculations performed agree with the encapsulation of a single {Na–OH_2_}^+^ moiety; structures containing a sodium cation and two or no water molecules were found at higher Gibbs energies (19.2 and 15.5 kcal mol^–1^, respectively). The encapsulation of a dinuclear {Na_2_OH_2_}^2+^ unit in the cubic cavity also produces a less stable complex (+7.0 kcal mol^–1^).

**Figure 8:**
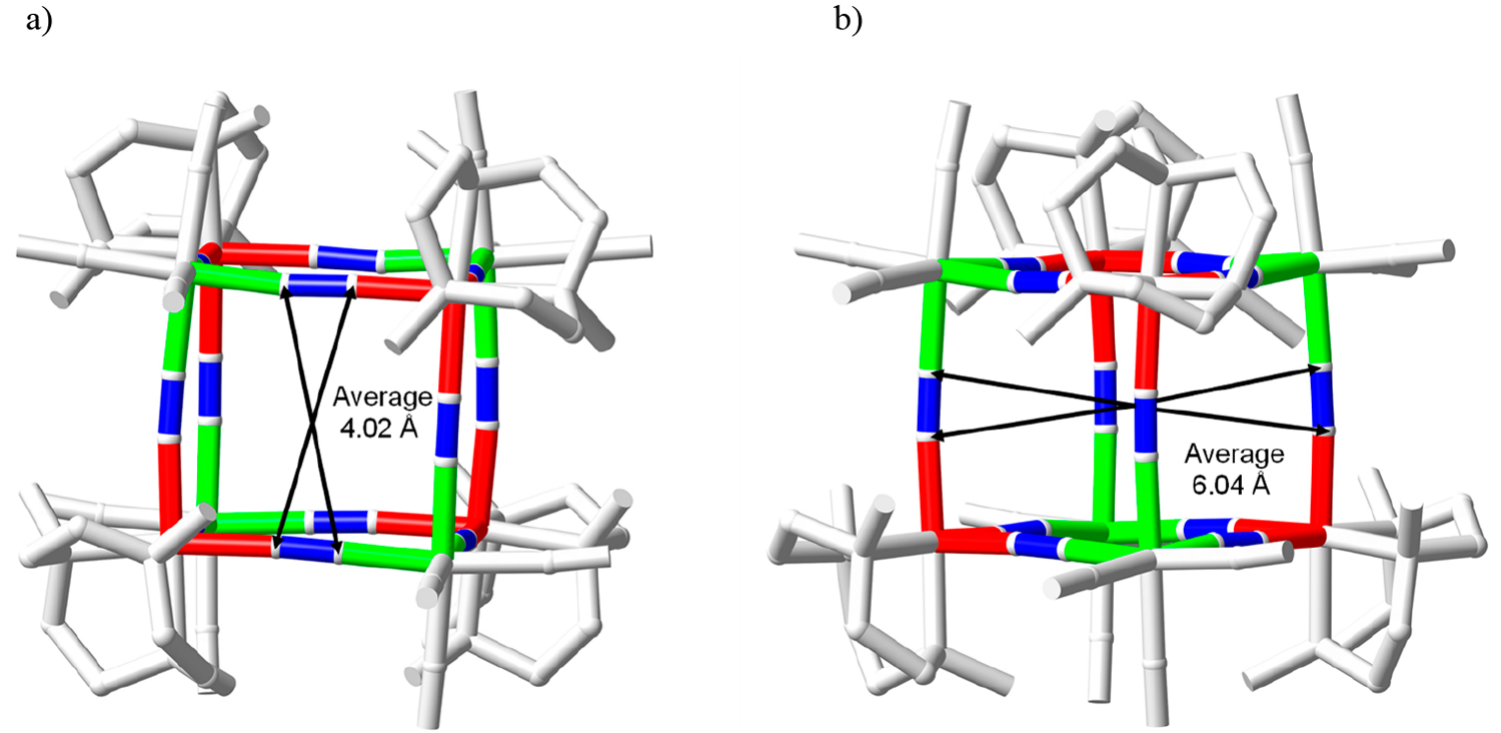
Relevant portal (a) and cavity (b) dimensions of the cubic cage of the Na_3_{{Na–OH_2_}⊂[{Co^III^(Me_3_-tacn)}_4_{Fe^II^(CN)_6_}_4_]}·22H_2_O compound.

The relevant maximum dimensions for the square portal and cavity are also indicated in Figure 8, where the radii of the carbon (0.60 Å) and nitrogen (0.54 Å) atoms have been used, as indicated above. Likewise, for the structure of the lithium counterpart, a sphere of a maximum radius of ∼2 Å could go through the square portal of the cube and one of ∼3 Å could be set inside.

**Figure 9:**
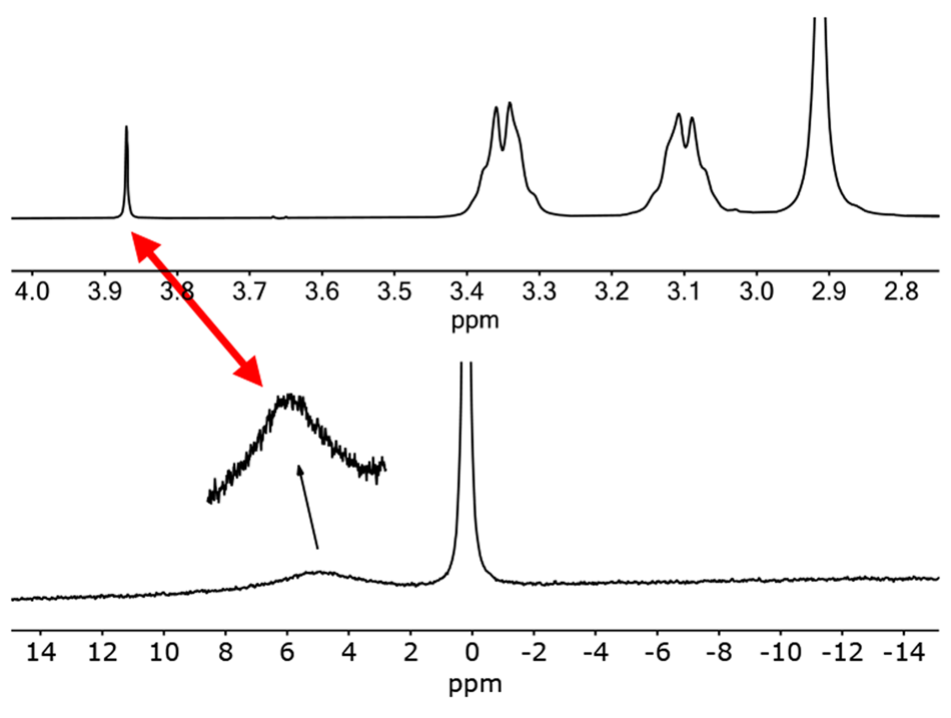
^1^H (top) and ^23^Na (bottom) NMR spectra of the Na_3_{{Na–OH_2_}⊂[{Co^III^ (Me_3_-tacn)}_4_{Fe^II^(CN)_6_}_4_]}·22H_2_O crystals dissolved in D_2_O.

The ^1^H and ^23^Na NMR spectra of these crystals in D_2_O confirm the formulation established by the XRD studies, as well as the inert character of the sodium ion and water molecule confined inside the cubic cage. It should be noted (Figure 9) that the ^23^Na NMR spectrum shows a broad signal for the ^23^Na nucleus of the confined unit. This is expected from the value of its quadrupole moment, its noncubic symmetry, and its likely fast tumbling inside the {{Co^III^(Me_3_-tacn)}_4_{Fe^II^(CN)_6_}_4_} cage (producing a sharp ^1^H NMR resonance for the {Na–OH_2_}^+^ unit at 3.87 ppm).

### Potassium Salt

The general procedure for the preparation and characterization of the potassium salt of the [{Co^III^(Me_3_-tacn)}_4_{Fe^II^(CN)_6_}_4_]^4–^ complex has already been described.^45^ The compound was found to be prevalent upon manipulation of the sodium salt samples, as expected from the exchange processes indicated in the next section. This prevalence (especially in ESI-MS procedures) is due to the unavoidable presence of potassium ions at micromolar concentration levels, such as those used for manipulating the complexes.^59^ This fact precluded the use of high-resolution ESI-MS as a reliable characterization technique of the compounds described in this work.

The absence of a water molecule inside the cubic cavity is confirmed by DFT calculations. In this case, the species encapsulating a simple K^+^ cation is more than 15 kcal mol^–1^ lower in Gibbs energy than the structure with an encapsulated {K–OH_2_}^+^ unit. This fact also indicates that larger alkali-metal cations, i.e., Rb^+^ and Cs^+^, if encapsulated, would produce structures with the metal alone within the cubic structure.

### Rubidium Salt

**Figure 10:**
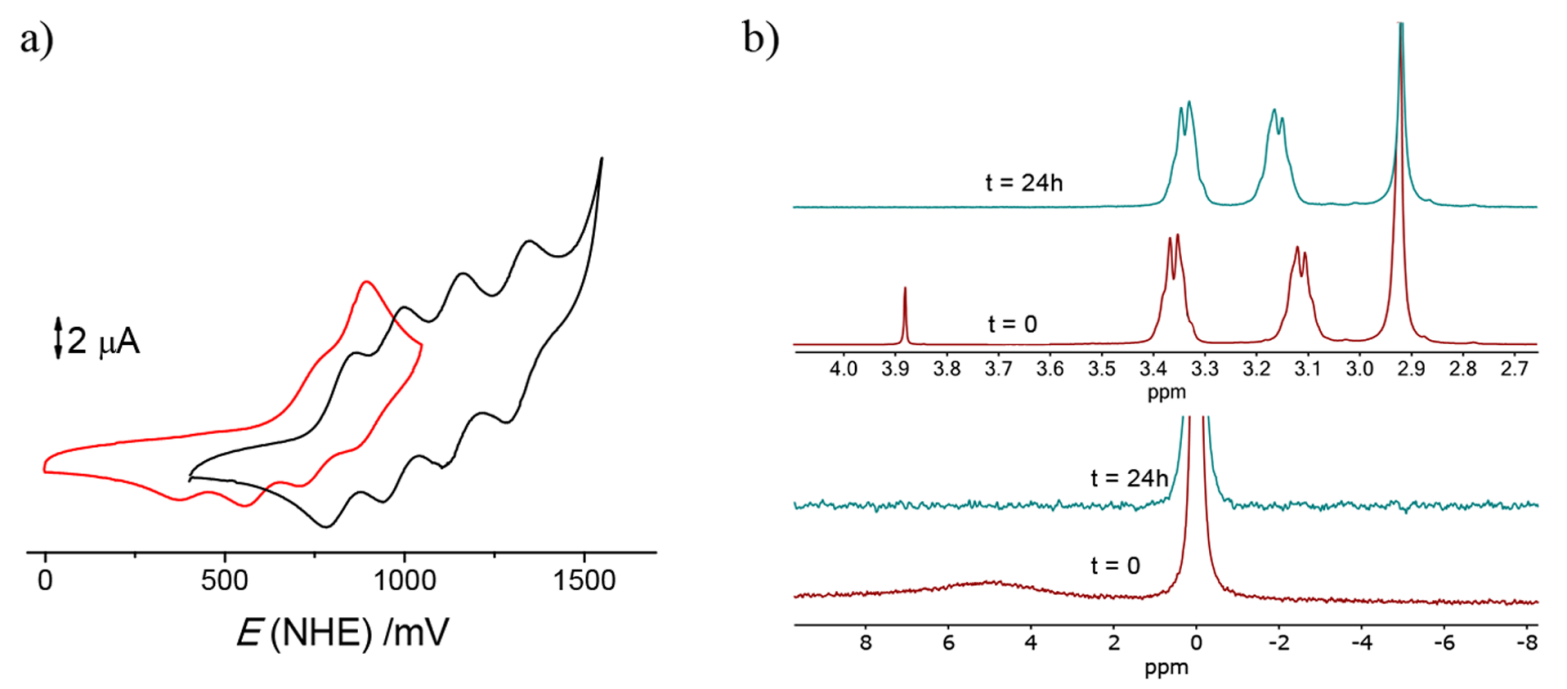
(a) Changes in the cyclic voltammogram of a 1 × 10^–3^ M solution of the sodium salt of the [{Co^III^(Me_3_-tacn)}_4_{Fe^II^(CN)_6_}_4_]^4–^ cube (red^45^) in a 0.20 M RbCl solution after 24 h (black). (b) Changes in the ^1^H (top) and ^23^Na (bottom) NMR spectra of the same species at 1 × 10^–2^ M in 1.0 M RbCl.

The corresponding Rb^+^ salt was obtained *in situ* by solution cation exchange (50–100-fold excess in order to have reasonable exchange times; see the next section) of the sodium salt. The final exchanged solution was subsequently characterized by NMR, electrochemistry, ICP-OES, and UV–vis spectroscopy (Figure S2). The absence of any encapsulated water in the cube is confirmed, as found for the potassium salt, by the absence of signals in the 3.5–4.5 ppm region of the ^1^H NMR spectrum; no differences were obtained when the spectrum was collected in a 0.1 M RbCl solution. Figure 10 collects the changes observed in the aqueous solution of the Na_3_{{Na–OH_2_}⊂[{Co^III^(Me_3_-tacn)}_4_{Fe^II^(CN)_6_}_4_]}·22H_2_O compound after 24 h by electrochemistry and ^1^H and ^23^Na NMR.

When the sample was cation-exchanged by Sephadex DEAE A-25 anionic chromatography in order to eliminate all “external” rubidium cations via 0.25 M LiCl elution, ICP-OES metal analysis indicated the absence of rubidium in the sample. Furthermore, the UV–vis spectrum obtained after this chromatographic procedure corresponded to the cubic structure referred to as void in the synthesis of the lithium derivative (Figures 2 and S2). That is, using anionic Sephadex DEAE A-25 chromatography, the confined rubidium cations are eliminated during workup (see the Adsorption Cation-Exchange Study section).

### Cesium Salt

The preparation of the cesium salt of the {{Co^III^(Me_3_-tacn)}_4_{Fe^II^(CN)_6_}_4_} cubic structure was tried *in situ* using the methodology indicated for the rubidium salt (see above). The process proved that, even after 2 weeks, the signals of “confined” water and sodium in the ^1^H and ^23^Na NMR spectra do not diminish in intensity; the substitution by cesium of the {Na–OH_2_}^+^ unit is thus not occurring.

### Solution Cation-Exchange Kineticomechanistic Study

In view of the important differences observed (with respect to the confinement and exchange of alkali cations and water in its cavity) in the behavior of the cubic {{Co^III^(Me_3_-tacn)}_4_{Fe^II^(CN)_6_}_4_} structures prepared, we decided to study from a kineticomechanistic perspective the exchange processes at variable concentrations, ionic strengths, temperatures, and pressures. In our previous report,^45^ we had already shown that the K^+^ for {Na–OH_2_}^+^ exchange study is feasible because of significant and reliable UV–vis spectral changes. All processes involved with the species prepared, as seen in Figures 3a and S2b, are observed to be quite sensitive to UV–vis spectral changes. Interestingly, although the confined sodium to potassium to rubidium exchange has been observed in an irreversible way, the known {{K}⊂[{Co^III^(Me_3_-tacn)}_4_{Fe^II^(CN)_6_}_4_]} cage^45^ has not been observed to undergo exchange to a rubidium species. Furthermore, the sodium-to-lithium exchange has not been observed, with prevalence in the ^1^H NMR of the signal of the {Na–OH_2_}^+^ units upon solution (D_2_O) of the sample in 0.1 M LiCl.

**Figure 11:**
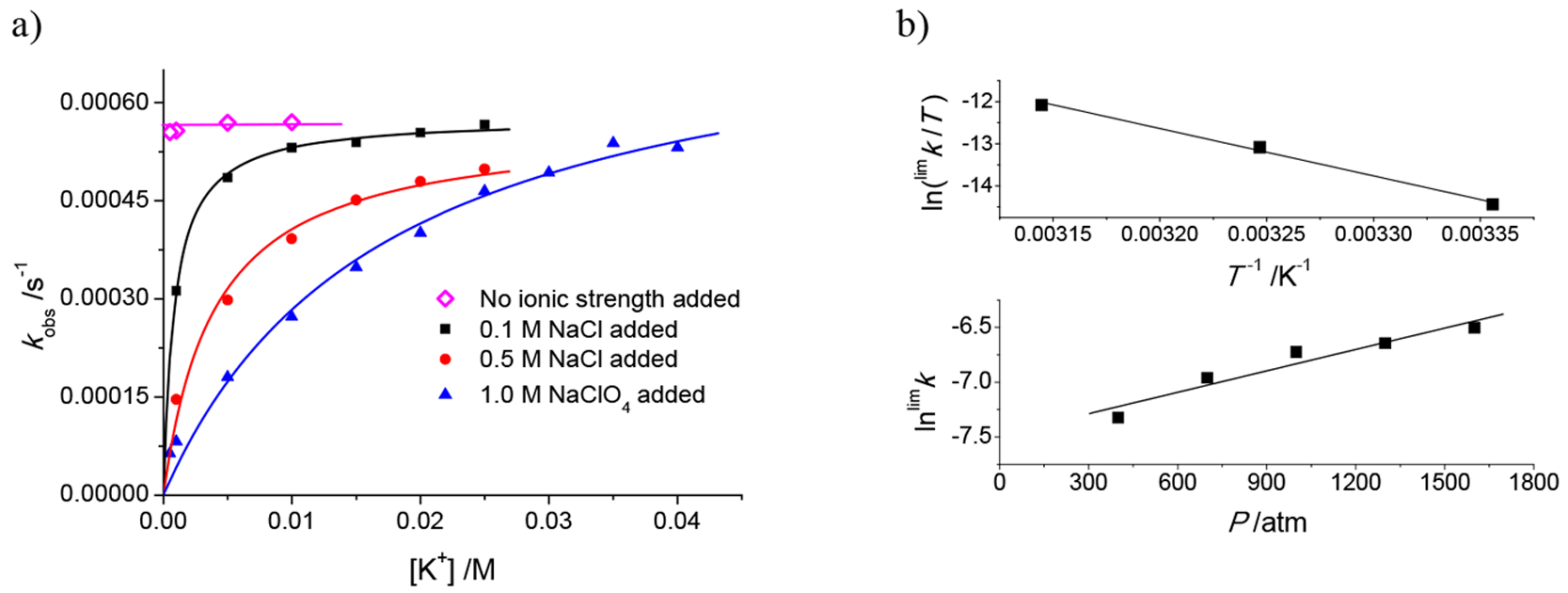
(a) [K^+^] dependence at 35 °C in aqueous solution of the values of *k*_obs_ for exchange from the sodium salt of the [{Co^III^(Me_3_-tacn)}_4_{Fe^II^(CN)_6_}_4_]^4–^ cubic cages. (b) Standard Eyring (top) or ln *k* versus *P* (bottom) plots for changes of the value of ^lim^*k* for the sodium-to-potassium cation-exchange process with temperature and pressure.

The preliminary results obtained for the {{Na–OH_2_}⊂[{Co^III^(Me_3_-tacn)}_4_{Fe^II^(CN)_6_}_4_]}-to-{{K}⊂[{Co^III^(Me_3_-tacn)}_4_{Fe^II^(CN)_6_}_4_]} exchange process have been extended to different conditions of temperature, pressure, and ionic strength to achieve the comprehensiveness of the studies. As was already indicated,^45^ the exchange process is not in equilibrium under pseudo-first-order (excess entering cation) conditions. Furthermore, a solution of the {{K}⊂[{Co^III^(Me_3_-tacn)}_4_{Fe^II^(CN)_6_}_4_]} species in 0.5 M NaCl does not produce any differences in the electronic spectrum, thus indicating the irreversibility of the exchange. The observed rate constants obtained for the exchange reaction are found to be independent of the entering cation concentration, at high concentration levels (0.050–0.10 M KCl) or low ionic strengths, as seen in Figure 11, which shows the effects observed on the values of *k*_obs_ upon variation of the values of [K^+^] and *I* ([Na^+^]). The data fit with a standard Eigen–Wilkins mechanism (Scheme S1) and rate law of the type indicated in eq 1a,^60−62^ with *K*_OS(K,Na)_ corresponding to a mixed outer-sphere interaction of the alkaline cations with an anionic cubic structure.
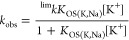
1a
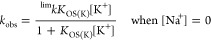
1b

For the experiments run in the absence of any other added cations (experiments having a neat *K*_OS(K)_ equilibrium constant, eq 1b), the outer-sphere interactions are very large, leading to *k*_obs_ = ^lim^*k* and favoring a limiting-exchange process.^61^ From these limiting values of *k*_obs_ (i.e., those measured at [KCl] = 0.10–0.20 M with no ionic strength added, according to Figure 11a) at different temperatures and pressures, the corresponding thermal and pressure activation parameters can be obtained using the standard Eyring (Figure 11b, top) or ln *k* versus *P* plots (Figure 11b, bottom).

**Figure 12:**
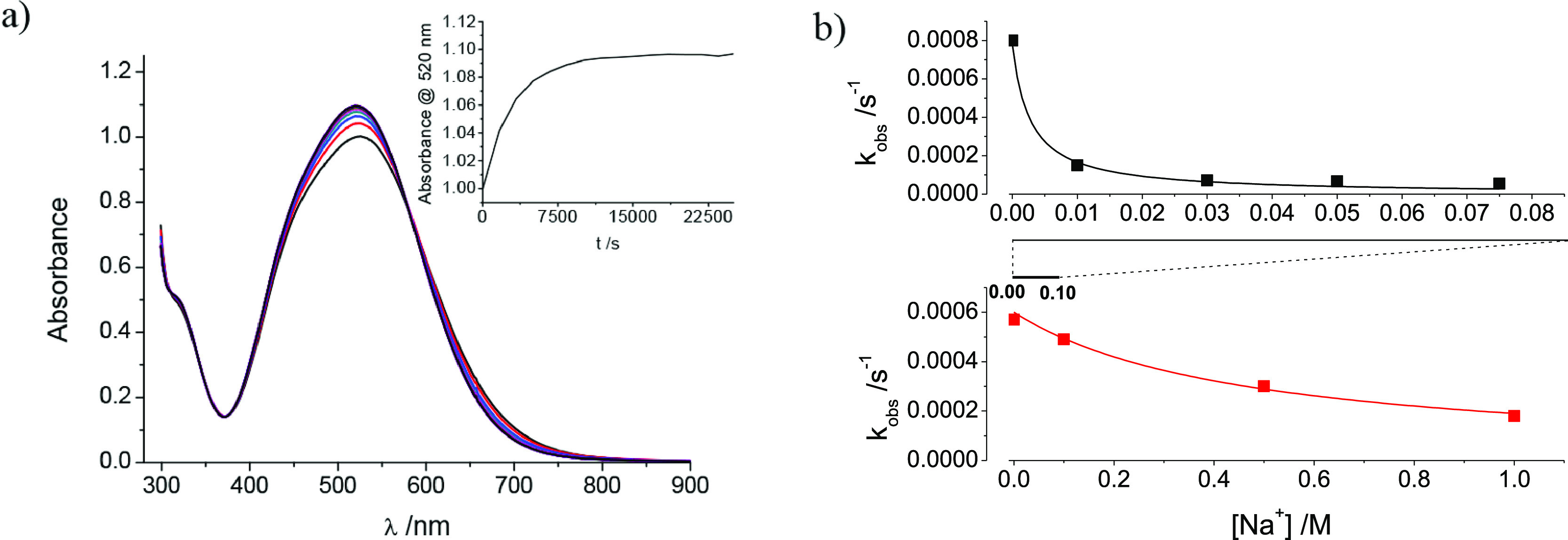
(a) UV–vis spectral changes observed upon reaction of a 2 × 10^–4^ M aqueous solution of the sodium salt of the [{Co^III^(Me_3_-tacn)}_4_{Fe^II^(CN)_6_}_4_]^4–^ cubic cage with RbCl (0.15 M) at 25 °C. (b) Effect of [Na^+^] added on the value of the observed rate constants for the confined cation exchange at [[{Co^III^(Me_3_-tacn)}_4_{Fe^II^(CN)_6_}_4_]^4–^] = 1 × 10^–4^ M: (top) {NaOH_2_}^+^ to Rb^+^ at 0.15 M RbCl (33 °C); (bottom) {NaOH_2_}^+^ to K^+^ at 0.005 M KCl (35 °C). Note the 10-fold difference in the [Na^+^] concentration scale.

It is clear that, because the *K*_OS(K,Na)_ equilibrium constant is only apparent at high [Na^+^] levels (and low [K^+^] values), the mixed outer-sphere species slows down the exchange.^63,64^ This effect could be simplistically associated with the presence of dead-end outer-sphere interactions with the Na^+^ cations, producing an alternative approximate rate law, such as that indicated in eq 2a.
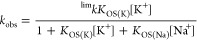
2a
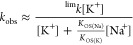
2bEquation 2b is a simplification of eq 2a for 1 ≪ (*K*_OS(K)_[K^+^] + *K*_OS(Na)_[Na^+^]), which applies in our case, enabling thus the determination of a simplistic (*K*_OS(Na)_/*K*_OS(K)_) ratio at a given [K^+^] and varying [Na^+^] values (Figure 12b, bottom). This value represents a measure of the productiveness of the outer-sphere precursor at different [Na^+^] values; its values are also collected in Table 1, together with the relevant kinetic and activation parameters for the process.

**Table 1:** Relevant Kinetic and Thermal and Pressure Activation Parameters for Exchange Reactions of the Alkaline Cations Studied on the [{Co^III^(Me_3_-tacn)}_4_{Fe^II^(CN)_6_}_4_]^4–^ Cubic Cages

exchange	medium	^app^(*K*_OS(medium)_/*K*_OS(entering)_)	10^4^ ^lim^*k* /s^–1^ (298 K)	Δ*H*^⧧^/kJ mol^–1^	Δ*S*^⧧^/J K^–1^ mol^–1^	Δ*V*^⧧^/cm^3^ mol^–1^
{Na–OH_2_}^+^ to K^+^	0.10–1.0 M [Na^+^]	*K*_OS(Na)_/*K*_OS(K)_ = 0.10 at 0.05 M KCl	1.7	94 ± 7	–4 ± 22	–16 ± 3
	0.10 M [H^+^]	*K*_OS(K)_ = 40 M^–1^ in these conditions	190	51 ± 4	–109 ± 13	a
{Na–OH_2_}^+^ to Rb^+^	0.010–0.10 M [Na^+^]	*K*_OS(Na)_/*K*_OS(Rb)_ = 50 at 0.15 M RbCl	2.7	75 ± 3	–64 ± 10	–22 ± 3
{Li–OH_2_}^+^ to {Na–OH_2_}^+^	0.0050–0.50 M [Na^+^]	*K*_OS(Li)_ = 30 M^–1^ at 0.050 M NaCl	0.63	64 ± 4	–113 ± 13	2.7 ± 0.6
{Li–OH_2_}^+^ to K^+^	0.050–1.0 M [Li^+^]	≈0^b^	0.33	93 ± 9	–21 ± 24	–16 ± 1
{Li–OH_2_}^+^ to Rb^+^	0.050–0.10 M [Li^+^]	≈0^b^	0.45	75 ± 5	–79 ± 15	–18 ± 2
{Li–OH_2_}^+^ to void		not applicable	0.032	92 ± 5	–44 ± 15	–26 ± 5

a Not determined because of a low solubility.

b No dead-end ion-pair effect observed.

Given the fact that the {{Na–OH_2_}⊂[{Co^III^(Me_3_-tacn)}_4_{Fe^II^(CN)_6_}_4_]} unit shows a well-established multiprotonation process with p*K*_a_ values of 2.3, 1.8, and 0.93^45^ and that outer-sphere interaction of the alkali cations with the anionic cubic structure seems to be dominant, the {Na–OH_2_}^+^ to K^+^ exchange process was also followed in an acidic medium. The acidity, nevertheless, had to be limited to 0.10 M HCl because of solubility issues (see previous section and the intensity of the UV–vis spectrum in Figure S3). Although the thermal activation parameters (collected in Table 1) were determined from the *k*_obs_ data at 0.10 M KCl (*k*_obs_ = ^lim^*k* according to Figure S3b), the activation volume could not be determined for the same solubility issues. Under these acidic conditions, the cubic structure is expected to be in a mixture of its di- and monoanionic (di- and triprotonated) forms, thus reducing considerably the value of *K*_OS(K)_, as observed in Figure S3b. Furthermore, the significant changes produced upon protonation in the cubic cage also translate on a faster exchange process for enthalpy reasons (Δ*H*^⧧^ being reduced to ca. half the value at pH 7) despite a less favorable entropy term.

The kineticomechanistic study of the cation interchange was further pursued by the Rb^+^ ion for {Na–OH_2_}^+^ exchange reaction, which had already been used for the preparation *in situ* of the rubidium salt (see the previous section). As for the K^+^ ion for {Na–OH_2_}^+^ exchange, the reaction showed well-behaved UV–vis spectral changes that allowed determination of the corresponding rate constants under pseudo-first-order conditions (Figure 12a). Furthermore, when the exchange experiments were conducted in the presence of varying concentrations of NaCl, again a definite slowing-down effect was observed. In fact, the exchange process becomes too slow to be measured at ca. [NaCl]_added_ > 10[RbCl] (Figure 12b, top), being [NaCl] > 150[KCl] for the K^+^ ion for {Na–OH_2_}^+^ exchange (Figure 12b, bottom). Even so, the absorbance changes are independent of the amount of NaCl added, indicating that no equilibrium is established. It is thus clear that the same competition between productive and dead-end outer-sphere complexes, determined for the K^+^ ion for {Na–OH_2_}^+^ exchange, is taking place in this case.

The values of *k*_obs_, determined under pseudo-first-order conditions and in the absence of any NaCl added (Table S1), are found to be independent of the [RbCl] within the 0.020–0.50 M range (at a 5 × 10^–5^ M concentration level of the cubic cage). These correspond to the ^lim^*k* values indicated above (eq 1b at high entering cation concentrations) and were used for determination of the thermal and pressure activation parameters indicated in Table 1 and Figure S4. The values determined (Table 1) indicate that, although for this exchange the enthalpy demand diminishes with respect to that by potassium, the entropy demand increases considerably, as well as the contraction occurring upon going to the transition state.

When the same exchange experiments were carried out on the major component obtained in the preparation of the lithium salt of the [{Co^III^(Me_3_-tacn)}_4_{Fe^II^(CN)_6_}_4_]^4–^ cage (i.e., void, as seen previously), no reaction was observed with aqueous 0.25–0.50 M NaCl, KCl, or RbCl solutions. The UV–vis spectra remained unchanged for days despite the fact that significant intensity differences should be observed upon cation entering, according to Figure 3a; no changes were observed in the ^1^H NMR spectra either.

From that point, a study of the exchange of the {Li–OH_2_}^+^ units to other alkaline cations in the samples having been lithium-enriched by Sephadex DEAE A-25 chromatography of {{Na–OH_2_}⊂[{Co^III^(Me_3_-tacn)}_4_{Fe^II^(CN)_6_}_4_]} (see the next section) was also conducted for comparison with the data for the {Na–OH_2_}^+^-unit-containing [{Co^III^(Me_3_-tacn)}_4_{Fe^II^(CN)_6_}_4_]^4–^ cube. Experiments were initially run to monitor the loss of the encapsulated unit (i.e., from {Li–OH_2_}^+^ to void, as observed during the preparative procedures). Despite the small spectral changes (Figure S5a), the process could be followed by UV–vis spectroscopy in a very reproducible way. From variation of the values of *k*_obs_ versus temperature and pressure (Figure S5b), the values of the thermal and pressure activation parameters derived are collected in Table 1. The data agree very well with those in the ^1^H NMR experiments, indicated in the previous section, and are within the values expected from the process, with solvation of the exiting lithium cation (ordering and compression) playing a crucial role, in this case not compensated by changes in the entering cation.

The reactions of the exchange of the cage having {Li–OH_2_}^+^ inert units (i.e., {{Li–OH_2_}⊂[{Co^III^(Me_3_-tacn)}_4_{Fe^II^(CN)_6_}_4_]}) with K^+^ or Rb^+^ were also effective, as observed by UV–vis and ^1^H NMR spectroscopy (see Figure S6a for an example). Similarly to the {Na–OH_2_}^+^ exchange processes indicated before, the values derived for *k*_obs_ were found to be independent of the concentration of the entering cations in the 0.0050–0.10 M range (Table S1), and no concentration-related differences in the absorbance changes were observed, in agreement with the lack of an equilibrium situation. The data at a 0.10 M cation concentration have been used to determine the values of the activation parameters collected in Table 1 (see Figure S6b as an example). As was already observed for the replacement of {Na–OH_2_}^+^ by K^+^, the presence of Li^+^ cations at different concentrations does not hamper the exchange (Table S1), indicating that *K*_OS(K or Rb)_ ≫ *K*_OS(Li)_, thus changing eq 2b to *k*_obs_ ≈ ^lim^*k*. That is, ion pairing with lithium cations does not behave as a dead-end phenomenon for these processes, in contrast with the fact observed when Na^+^ was added to the {Na–OH_2_}^+^ exchange processes (Table 1).

**Figure 13:**
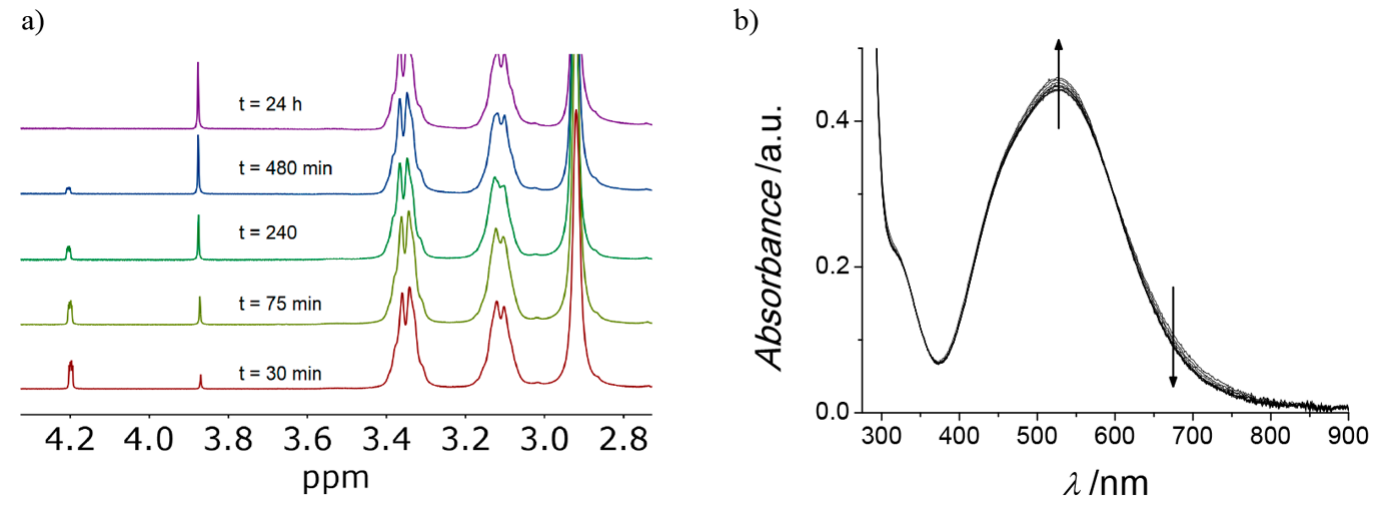
(a) Time-resolved ^1^H NMR changes observed upon solution of a sample of {{Li–OH_2_}⊂[{Co^III^(Me_3_-tacn)}_4_{Fe^II^(CN)_6_}_4_]} species in 0.10 M NaCl. (b) UV–vis spectral changes observed for a 5 × 10^–5^ M sample of the same compound with 0.10 M NaCl and 0.05 M LiCl at 35 °C.

Interestingly, the reaction of {{Li–OH_2_}⊂[{Co^III^(Me_3_-tacn)}_4_{Fe^II^(CN)_6_}_4_]} with NaCl showed distinct trends from those observed for the cations above:

(i) ^1^H NMR experiments at the ca. 2 × 10^–3^ M concentration level (>7 × 10^–3^ M in “external” Li^+^) showed a clear Li^+^-to-Na^+^ exchange, while keeping the accompanying encapsulated nondeuterated water molecule from the {M–OH_2_}^+^ units inside the cage (Figure 13a).

(ii) Although no reaction is observed on solutions of ca. 5 × 10^–5^ M in the {{Li–OH_2_}⊂[{Co^III^(Me_3_-tacn)}_4_{Fe^II^(CN)_6_}_4_]} cage (1.5 × 10^–4^ M in “external” Li^+^), a definite reaction is observed by UV–vis spectroscopy at 2 × 10^–4^ M cage concentration levels (>7 × 10^–4^ M in “external” Li^+^).

(iii) When the experiments at the 5 × 10^–5^ M cage concentration level were conducted with different concentrations of Li^+^ (LiCl or LiClO_4_), a definite reaction was observed by time-resolved UV–vis spectroscopy (Figure 13b). The time span agrees with the ^1^H NMR experiments, and the value of *k*_obs_ is found to decrease with decreasing LiCl concentration (Figure S7).

In all cases, no equilibrium was observed, and the values of *k*_obs_ were found to be independent of the concentration of Na^+^ or Li^+^ cations provided the limiting behavior indicated in Figure S7 is attained (see above). The results agree with an initial outer-sphere fast equilibrium reaction producing an {Li^+^;{{Li–OH_2_}⊂[{Co^III^(Me_3_-tacn)}_4_{Fe^II^(CN)_6_}_4_]} complex that represents the active species undergoing the exchange process quantified by eqs 1a and 2a, with *k*_obs_ ≈ ^lim^*k*. In this case, contrary to its sodium counterpart, the outer-sphere complex of the cage is not a dead-end species but instead the active species for the Li^+^-to-Na^+^ exchange within the {M–OH_2_}^+^ units. The values of *k*_obs_ at [LiCl] = 0.050 M and [NaCl] = 0.10 M have been used for derivation of the thermal and pressure activation parameters collected in Table 1. The value of the equilibrium constant leading to formation of the active precursor species [^app^(*K*_OS(medium)_/*K*_OS(entering)_)] is also collected in the table, where it is made clear that important differences exist in the process involved. The values of the enthalpy and entropy of activation are very distinct from the other exchange processes studied, with the exception of that on the protonated species of the {{Na–OH_2_}⊂[{Co^III^(Me_3_-tacn)}_4_{Fe^II^(CN)_6_}_4_]} structure (a situation rather similar to that of a tight outer-sphere complex with a Li^+^ cation). The sign of the value of the volume of activation is also reversed, indicating a small expansion upon going to the transition state, in contrast to the rather homogeneous contraction observed for the other reactions shown in Table 1.

### Adsorption Cation-Exchange Study

As indicated in the preparative section, the {{Rb}⊂[{Co^III^(Me_3_-tacn)}_4_{Fe^II^(CN)_6_}_4_]} unit loses its encapsulated Rb^+^ upon chromatography exchange with Li^+^ on a Sephadex DEAE A-25 column, and, consequently, the void cubic structure is obtained. In view of these results, we have conducted a comprehensive study on these heterogeneous processes occurring on the fully characterized lithium and sodium salts of the [{Co^III^(Me_3_-tacn)}_4_{Fe^II^(CN)_6_}_4_]^4–^ cubic cages.

Upon loading of an aqueous solution of the Na_3_{{Na–OH_2_}⊂[{Co^III^(Me_3_-tacn)}_4_{Fe^II^(CN)_6_}_4_]}·22H_2_O complex on a Sephadex DEAE A-25 anion-exchange column, a single very well-defined and very narrow band is held on the top (as would be expected from its large negative charge). After thorough washing with water, elution was commenced with 0.1 M NaClO_4_; the band started to move rather sluggishly, and the concentration of the eluent was increased to 0.3 M. The purple solution thus obtained was quickly taken to dryness at room temperature and thoroughly washed with acetone to eliminate most of the free NaClO_4_. The ^1^H NMR spectrum of this sample shows the signal of the confined water molecule at 3.87 ppm with an intensity that is identical with that of the initial sample, indicating that under these conditions the {Na–OH_2_}^+^ units are not expelled from the cage.

**Figure 14:**
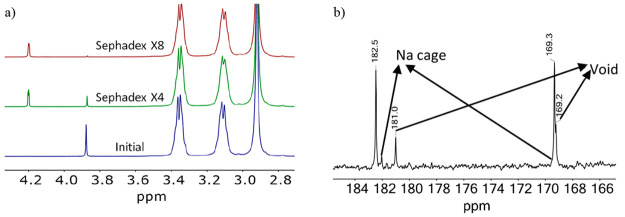
(a) ^1^H NMR spectral changes observed after four and eight repetitions of Sephadex DEAE A-25 LiClO_4_-eluted chromatography of a sample of the sodium salt of the [{Co^III^(Me_3_-tacn)}_4_{Fe^II^(CN)_6_}_4_]^4–^ cubic cage (see the text). (b) ^13^C NMR spectrum of the intermediate sample (Sephadex × 4).

When the same experiment was conducted by elution with LiClO_4_, the final sample showed a ^1^H NMR spectrum with a less intense signal at 3.87 ppm (from the confined {Na–OH_2_}^+^ units). Furthermore, the appearance of a quadruplet at 4.21 ppm indicated that encapsulated {Li–OH_2_}^+^ units were present, which is in absolute contradiction with the behavior observed in a homogeneous aqueous solution. After a 10-fold dilution, the loading/elution process was repeated three more consecutive times, and the {Na–OH_2_}^+^-to-{Li–OH_2_}^+^ unit exchange is observed to have occurred up to 75% (Figure 14a). The ^13^C NMR spectrum is even more revealing; when compared with that in Figure 2b, the signals of the sodium cage are practically not observed, while the signals of the void cubic structure are apparent because of the long acquisition times required for a reliable spectrum, during which some {Li–OH_2_}^+^-to-void exchange occurred (see the previous section). Enrichment of the sample by repeating the procedure indicated above four more times produced up to a 9:1 ratio of cages encapsulating either the {Li–OH_2_}^+^ or {Na–OH_2_}^+^ units, respectively. From the final sample, XRD-quality crystals were obtained and analyzed (Figure 4).

The crude compound obtained from the preparation of the lithium salt of the [{Co^III^(Me_3_-tacn)}_4_{Fe^II^(CN)_6_}_4_]^4–^ cube was studied in a similar way. In this case, again the signal of the residual {Na–OH_2_}^+^ units present in the crude product disappears upon LiClO_4_ elution from a Sephadex DEAE A-25 column, while elution with NaClO_4_ produces a solution without the signal of the encapsulated {Li–OH_2_}^+^ unit. It is not clear whether this latter effect is due to the heterogeneous reaction on the adsorbed sample or the simple “emptying” of the lithium {Co^III^(Me_3_-tacn)}_4_{Fe^II^(CN)_6_}_4_} cubic cage upon standing, as indicated in the previous preparative and solution-exchange sections.

## Discussion

### Compounds

The preparation and isolation of the lithium, sodium, and potassium salts of the cube indicated in Chart 1 have been achieved by the self-assembly of {Co^III^-NC-Fe^II^} edge units generated from the outer-sphere redox/labile substitution/inner-sphere redox processes known for some time and that we have comprehensively developed.^41,55,65^ The presence of the {Co^III^-NC-Fe^II^-CN-Co^III^} units can also be achieved from the same processes, but the prevalence of trans isomerism of the iron(II) hexacyanido unit would not allow the formation of a cubic structure, which requires a *fac* isomeric assembly.^42^ In this respect, the formation of tetranuclear *mer*-{(Co^III^-NC)_3_-Fe^II^} assemblies has also been reported.^43^ Nevertheless, its formation necessitates substitution processes on the inert Co^III^ centers because of both the dramatic increase in the *E*^0^(Fe^III^/Fe^II^) redox potential and the need for a (2+)([Co^III^-NC-Fe^II^-CN-Co^III^})/(3+){Co^III^} outer-sphere contact.^43^ The final self-assembly process from the {Co^III^-NC-Fe^II^} edge units is clearly evidenced by the absence of measurable amounts of any {(Co^III^-NC)_2_-Fe^II^} or {Co^III^(-NC-Fe^II^)_2_} species in solution, even when large excesses of iron or cobalt building blocks are used.^45^ Clearly the final assembly process is driven by thermodynamics and the relatively less inert character of the ancillary monodentate ligands on the initial {Co^III^(Me_3_-tacn)} building block unit.^66^

Characterization of the compounds has been achieved using standard techniques. ^7^Li and ^23^Na NMR and ^1^H DOSY NMR experiments also provided clear evidence of the confined character of alkali-metal ions and the associated slowly exchanging water molecule for the sodium and lithium species. Electrochemical experiments for the structures having a tumbling {M–OH_2_}^+^ unit inside show irreversible behavior, which is not present for the simpler void, K^+^- and Rb^+^-containing structures, where four fully reversible and independent iron-centered redox processes are observed, similar to those for other units of the same family of compounds.^44,46,47,52,54,67^ Clearly, the distinct oxidation of the Fe^II^ units seems to be highly affected by the presence of dynamically distributed {M–OH_2_}^+^ entities inside the cube. As for the XRD data, the bond distances are the expected according to all of the data available. The Co^III^-NC-Fe^II^ distance is within the 4.90–4.94 Å range observed for all of the di- and trinuclear structures indicated in Chart 1 and slightly larger than that observed for the protonated {(Co^III^)_2_(-NC-Fe^II^)_2_} square structure (4.83 Å). The intermetallic distances are effectively shorter than the Fe^III^-NC-Fe^II^ distance observed in Prussian Blue (5.07 Å),^68^ as expected from the differences in the electronic nature between the Fe^III^ and Co^III^ centers.

It is also important to mention the preparative origin of the internal cage-encapsulated units (despite the substitution processes observed); in all cases, the kinetically controlled assembled structures are generated by encapsulating the cations available in the assembly medium. The stability of the structure containing the {Na–OH_2_}^+^ units in comparison with that containing the equivalent {Li–OH_2_}^+^ entities is remarkable because the sodium form is found even if only residual quantities of sodium are present in the reaction medium (see the preparation of the lithium salt). Furthermore, the structure with encapsulated {Li–OH_2_}^+^ units tends to lose its guest to the medium, thus providing the void structure. In the same line, the surprising fact that the lithium inner cations are exchanged to sodium while maintaining the confined water^57^ indicates the structural stabilizing importance of the encapsulated water molecule (already observed in the electrochemical experiments) for these small alkali-metal cations.

The void cubic structure detected upon dissociation of the {Li–OH_2_}^+^ units from the lithium-assembled species merits some discussion. The cavity size of the structures indicated in Figures 4 and 7 clearly shows that an empty possibility is not feasible. Although the possible existence of encapsulated water clusters inside of the cubic structure cannot be ruled out,^11,69^ proton exchange with the deuterated D_2_O solvent makes them undetectable by ^1^H NMR. Nevertheless, the absence of any positive charge inside a highly negative cavity would point to a labile exchange of any Lewis base such as water.

### Aqueous Cation Exchange

With respect to the kineticomechanistic study of the exchange reactions in solution of the encapsulated units inside the [{Co^III^(Me_3_-tacn)}_4_{Fe^II^(CN)_6_}_4_]^4–^ cube, three general aspects have to be considered from the data in Table 1: (i) the unobserved entry of alkaline cations inside the void cube; (ii) the irreversible exchange sequence observed; (iii) the determinant involvement of outer-sphere complexes in the processes.

On the one hand, the exchange processes seem to be only thermodynamically favored upon hydration of cations contained inside the cage structure. That is, the entry of hydrated alkaline cationic entities into void cubes is not occurring because it implies uncompensated dehydration of the entering units. Furthermore, in aqueous solution, only a one-way route, from smaller to larger alkali-metal cation, is observed, indicating that the more favored hydration of the exiting smaller encapsulated units in the cage plays a determining role. The substitution sequence does not operate starting from the potassium-containing cage, thus indicating the rather good energetic fitting of the cation inside the cage.

On the other hand, although outer-sphere ion pairing between 4– and 1+ charges in solution is expected (producing the observed limiting kinetics),^61,70,71^ the Na^+^-dead-end and Li^+^-activating selective pairing in the {Na–OH_2_}^+^ (to K^+^ and Rb^+^) and{Li–OH_2_}^+^ (to {Na–OH_2_}^+^) exchanges is remarkable. Clearly, the outer-sphere {Na^+^;{{Na–OH_2_}^+^⊂[{Co^III^(Me_3_-tacn)}_4_{Fe^II^(CN)_6_}_4_]^4–^}} pairs are stable and comprehensive enough (Table 1) to hamper the precursor pairing with K^+^ or Rb^+^, thus stopping the exchange process, with the effect being more important with the larger Rb^+^ species. For the {{Li–OH_2_}⊂[{Co^III^(Me_3_-tacn)}_4_{Fe^II^(CN)_6_}_4_]} architecture, the equivalent {Li^+^;{{Li–OH_2_}^+^⊂[{Co^III^(Me_3_-tacn)}_4_{Fe^II^(CN)_6_}_4_]^4–^} pairs do not hamper the reaction, activating the {Li–OH_2_}^+^-to-{Na–OH_2_}^+^ exchanges instead. Clearly, the nature of the precursor {Na^+^;Li^+^;{{Li–OH_2_}^+^⊂[{Co^III^(Me_3_-tacn)}_4_{Fe^II^(CN)_6_}_4_]^4–^}} does not correspond merely to a simple ion-pair complex. This is a fact that can be related with the small size of the lithium cation,^72,73^ which should parallel the behavior of {Li^+^;{{Li–OH_2_}^+^⊂[{Co^III^(Me_3_-tacn)}_4_{Fe^II^(CN)_6_}_4_]^4–^}} with the species obtained upon protonation of the cubic structure (see below). Similarly, specific kinetic runs on the {Na–OH_2_}^+^-to-K^+^ exchange were carried out in the presence of high ionic strength concentrations (ca. 1–3 M) of LiClO_4_. The rate constant values were about 1.5–2.0-fold higher than the values obtained for ^lim^*k* when Na^+^ or no ionic strength medium was used (Table S1). Undoubtedly, the effect of Li^+^ association with the {{Na–OH_2_}^+^⊂[{Co^III^(Me_3_-tacn)}_4_{Fe^II^(CN)_6_}_4_]^4–^} compound also activates the {Na–OH_2_}^+^-to-K^+^ exchange.

As for the kinetic and activation parameters collected in Table 1, they must be considered in groups, according to the discussion above. The simpler discussion corresponds to the formation of the void structure from the {{Li–OH_2_}⊂[{Co^III^(Me_3_-tacn)}_4_{Fe^II^(CN)_6_}_4_]} architecture. The value of the rate constant at 25 °C is one order of magnitude smaller than the rest of the exchanges, while the values of the enthalpy and entropy of activation are rather similar to the exchange by K^+^ and close to that by Rb^+^. Interestingly, the value of the volume of activation is much more negative, indicating the neat simple hydration of the existing {Li–OH_2_}^+^ units, thus compressing some of the external aqueous medium.

With respect to the {Li–OH_2_}^+^ to K^+^ and Rb^+^ exchange, the values of the rate constants are rather similar. The values of the thermal activation parameters show a Δ*H*^⧧^(K^+^) > Δ*H*^⧧^(Rb^+^) and Δ*S*^⧧^(K^+^) > Δ*S*^⧧^(Rb^+^) sequence, in accordance with higher energetic and disorder demands for the dehydration and entry of the smaller K^+^, compared with the less well-hydrated entering Rb^+^. The trend of the values of the activation volumes is in very good agreement with the entropy data. This reasoning parallels the trends arising from the exchange of {Li–OH_2_}^+^ by K^+^ and Rb^+^ on the {{Li–OH_2_}⊂[{Co^III^(Me_3_-tacn)}_4_{Fe^II^(CN)_6_}_4_]} nonprotonated architecture.

The two remaining entries in Table 1 are associated with rather different reaction starting materials. The reaction corresponds to {Na–OH_2_}^+^ exchange on either partially protonated (i.e., {{Na–OH_2_}^+^⊂[{Co^III^(Me_3_-tacn)}_4_{Fe^II^(CN)_6_}_4_H_(2 or 3)_]^(2– or −)^}),^45^ or tightly bound (i.e., {Li^+^;{{Li–OH_2_}^+^⊂[{Co^III^(Me_3_-tacn)}_4_{Fe^II^(CN)_6_}_4_]^4–^}}) ion-pair architectures. In both cases, the effective charge of the cubic cage should be considered less negative, which should enable a lower enthalpy, demanding a positive unit exiting process. In the same way, the values obtained for the activation entropy for these processes are more negative, indicating a higher-ordered transition state, which is accompanied by a surprisingly small expansion for the {Li–OH_2_}^+^-to-{Na–OH_2_}^+^ exchange. The combination of these two apparently opposite trends has been repetitively associated with a variety of reactions with the actuation of hydrogen-bonding and ordered solvent network interactions in the transition state.^74−76^ This fact agrees very well with the presence of positive charges (H^+^ or Li^+^) tightly bound to the [{Co^III^(Me_3_-tacn)}_4_{Fe^II^(CN)_6_}_4_]^4–^ cubic cage.

Finally, the aspects related to the {Na–OH_2_}^+^ by {Li–OH_2_}^+^ substitution in Sephadex DEAE A-25 chromatography have to be discussed in view of the formally thermodynamically uphill reaction in aqueous solution, where the inverse irreversible process is observed. The process observed results in extraction from the cage of {Na–OH_2_}^+^ units or Na^+^ cations that are replaced by the smaller lithium counterparts, with the final compound being the somehow elusive {Li–OH_2_}⊂[{Co^III^(Me_3_-tacn)}_4_{Fe^II^(CN)_6_}_4_]} structure, obtained only as a minor product during preparative procedures. Adsorption of the highly charged {{Na–OH_2_}⊂[{Co^III^(Me_3_-tacn)}_4_{Fe^II^(CN)_6_}_4_]} species on the protonated (diethylamino)ethyl positively charged bead results in expulsion of the encapsulated sodium. From that point, the smaller lithium cations enter the cubic-cage-adsorbed architecture (which is not observed in solution). The unobserved entry of the same species into the void [{Co^III^(Me_3_-tacn)}_4_{Fe^II^(CN)_6_}_4_]^4–^ Sephadex-immobilized species can be directly related to a probable smaller size of the portal in such a complex, as seen in the trends in Figures 5 and 8. A similar anchoring process on acidic surfaces has been observed for these types of complexes,^53^ and, more recently, the expansion/compression of some helicoidal structures has been detected in the solution/solid state, involving the expulsion of some encapsulated cations.^77^

## Conclusions

The redox-triggered assembly reaction of cubic mixed-valence cyanide-bridged Co^III^/Fe^II^ compounds (PBAs) can be achieved using different alkali-metal cations that do not act innocently as countercations of the highly negative units assembled. The complexes formed confined {M–OH_2_}^+^ or M^+^ units in a rather inert manner, depending on the size of the metal ions involved, which makes the cubes hosts for these cation guests. The compound with an encapsulated {Na–OH_2_}^+^ unit is highly resistant in rather extreme acidic and basic aqueous media but can be exchanged with a K^+^ cation, which seems to be the thermodynamic well of the cation-exchange processes. The equivalent complex with confined {Li–OH_2_}^+^ loses its guest to the bulk solution upon prolonged standing, producing a nonreactive void {Co^III^(Me_3_-tacn)}_4_{Fe^II^(CN)_6_}_4_} structure. Electrochemistry and ^1^H NMR spectroscopy represent excellent tools for the distinction between confined {M–OH_2_}^+^ or M^+^ units.

The noninnocent behavior of the countercations in the chemistry involved is also evident when exchange reactions of the confined cationic units are considered. The formation of ion-pair complexes between the highly negatively charged cubic units and Na^+^ produces dead-end blocking of the exchange reactions, while for Li^+^ ion pairing, the substitution process is accelerated, similar to that for partial protonation of the cubic species.

The ligand nature of the cages can be studied kineticomechanistically via the exchange processes of their different alkali-metal salts. The processes show an energetic signature that involves the differences between the hydration of the entering and leaving cations, as indicated by the differences in the entropy and volume of activation. The strongly hydrogen-bonded character of the water inside the cage is indicated by both its lower acidity (as seen by ^1^H NMR) and the fact that lithium-to-sodium exchange occurs while maintaining the same confined water (as seen by ^1^H NMR in D_2_O). The portal dimensions of the cage (as determined from XRD) also clearly explain the fact that no exchange to Cs^+^ is observed in solution despite the persistent presence of encapsulated Cs^+^ cations in PBAs.

The surprising uphill selective reaction from {{Na–OH_2_}⊂[{Co^III^(Me_3_-tacn)}_4_{Fe^II^(CN)_6_}_4_]} to {{Li–OH_2_}⊂[{Co^III^(Me_3_-tacn)}_4_{Fe^II^(CN)_6_}_4_]} (not occurring from the void {{Co^III^(Me_3_-tacn)}_4_{Fe^II^(CN)_6_}_4_}) on samples adsorbed on a Sephadex DEAE A-25 column has to be explained by the immobilization of an expanded {{Na–OH_2_}⊂[{Co^III^(Me_3_-tacn)}_4_{Fe^II^(CN)_6_}_4_]} species, which allows the entry of a flooding amount of Li^+^_aq_ used for elution. This methodology opens up possibilities for the reversible entry of lithium cations in other structures.

## Experimental Section

### Physical Methods

The ^1^H and ^13^C NMR spectra were recorded on a Bruker 400Q or a Bruker 500 spectrometer at 25 °C at the Unitat de RMN d’Alt Camp de la Universitat de Barcelona, and the ^23^Na and ^7^Li NMR spectra were recorded on a Bruker 500 instrument. DOSY NMR measurements were performed on a Bruker 400 MHz NMR spectrometer (see the Supporting Information).

ICP-OES and ICP-MAS was also carried out at the Centres Científics i Tecnològics (Universitat de Barcelona) on a PerkinElmer Optima instrument. UV–vis spectra were recorded on a HP5483 or a Cary 50 instrument. IR spectra were recorded on a Thermo Scientific Nicolet iS5 FT-IR instrument using an ATR system.

Electrochemistry experiments were carried out at 25 °C and 100 mV s^–1^ with a BioLogic SP-150 instrument. A glassy carbon working electrode, a Ag/AgCl (saturated KCl or NaCl) reference electrode, and a platinum wire counter electrode were used in 1 × 10^–3^ M solutions of the sample with a 0.1 M chloride (or perchlorate) cage cation as the supporting electrolyte. All potentials are given versus the normal hydrogen electrode, once corrected for the reference electrode used.

The kinetic profiles for the reactions at atmospheric pressure were followed by UV–vis spectroscopy in the 900–300 nm range on a Cary 50 or an Agilent HP8453A instrument equipped with thermostated multicell transports. For runs at elevated pressure, the previously described high-pressure setup^74,78,79^ was used for connection to a J&M TIDAS S300 instrument.

### X-ray Structure Analysis

For the lithium derivative of the cubic structure, a black prismlike specimen of C_60_H_110_Cl_5_Co_4_Fe_4_Li_9_N_36_O_33_, Li_8_{{LiOH_2_}⊂[{Co^III^(Me_3_-tacn)}_4_{Fe^II^(CN)_6_}_4_]}(ClO_4_)_5_·12H_2_O, with approximate dimensions of 0.180 × 0.130 × 0.130 mm, was used for XRD analysis. For the sodium derivative of the cubic structure, a black prismlike specimen of C_60_H_130_Co_4_Fe_4_N_36_Na_4_O_23_, Na_3_{{NaOH_2_}⊂[{Co^III^(Me_3_-tacn)}_4_{Fe^II^(CN)_6_}_4_]}·22H_2_O, with approximate dimensions of 0.180 × 0.270 × 0.460 mm, was used for analysis. The X-ray intensity data were measured on a D8 Venture system equipped with a multilayer monochromator and a molybdenum microfocus (λ = 0.71073 Å; see the Supporting Information for details).

### Materials

Compound [Co(Me_3_-tacn)Cl_3_] was prepared according to literature methods.^66^ Na_4_[Fe^II^(CN)_6_] and K_4_[Fe^II^(CN)_6_] were recrystallized twice from the commercially available material before use. Li_4_[Fe^II^(CN)_6_] was prepared by a modification of the published procedure.^80^ An aqueous solution of K_4_[Fe^II^(CN)_6_] was treated with an excess of a solution of LiClO_4_ (3-fold); after cooling for 2 days at 3–4 °C; the resulting solution was filtered and taken to dryness at 50 °C. The solid obtained was thoroughly washed with ethanol and the remaining off-white solid collected. IR: ν_(stretch CN)_ 2123, 2040 cm^–1^

All of the other commercially available chemicals were of analytical grade and were used as received.

### Compounds

The preparation and isolation of all of the different solid salts of the [{Co^III^(Me_3_-tacn)}_4_{Fe^II^(CN)_6_}_4_]^4–^ cube were carried out by the same procedure that has already been described.^45^

Briefly, to an 0.01 M aqueous suspension of [Co(Me_3_-tacn)Cl_3_] at pH 7–8 was slowly added a 0.07 M solution of the corresponding salt of the [Fe^II^(CN)_6_]^4–^ anion in a 2–3-fold excess (also at pH 7–8). The resulting mixture became very dark and, after allowed to stir overnight at 40–50 °C, was filtered to eliminate any precipitated solids and loaded onto a Sephadex G-25 size exclusion chromatographic column (2 × 25 cm) in several aliquots. The retained dark band was eluted with water; an initial gray-blue polymer was discarded, while the central part of the remaining purple band was collected; a final trailing off-yellow band (an excess of hexacyanidoferrate(II)) was also discarded. The procedure was repeated twice. After concentration to a small volume at 40–50 °C, the remaining solution was left to evaporate to dryness in air and the final solid sample was analyzed by ICP (iron/cobalt ratio), ^1^H and ^13^C NMR, ^23^Na and ^7^Li NMR (when applicable), UV–vis and IR spectroscopy, and CV. The characterization data for the sodium and potassium salts fully agree with those described^45^ and are given in the Supporting Information, together with those of the new salts prepared.

The lithium and sodium salts of the [{Co^III^(Me_3_-tacn)}_4_{Fe^II^(CN)_6_}_4_]^4–^ cage could also be purified by Sephadex DEAE A-25 chromatography. A solution of the prepared compounds (*I* = 0.05 M) was loaded onto the columns and eluted with 0.2–0.3 M NaClO_4_ or LiClO_4_. The relevant fraction was taken to dryness at room temperature and the solid obtained thoroughly washed with acetone to eliminate the remaining sodium or potassium perchlorates. The alternative cross-purification of the sodium salt by elution with LiClO_4_ produced a lithium salt of the cage with distinct properties.

### Computational Details

All of the DFT calculations were carried out using the *Gaussian09* (revision D.01)^81^ software. The hybrid functional PBE was employed^82,83^ for all of the calculations along with the def2svp^84,85^ basis set for all of the atom types. Ultrafine integration grids were used in all calculations to ensure a satisfactory convergence. In all cases, the solvation energies were computed in water with the IEF-PCM continuum dielectric solvation model^86,87^ using the SMD radii and nonelectrostatic terms.^88^ The dispersion energy correction terms were included by using the D3 method of Grimme.^89^ Vibrational analyses were performed for all of the computed structures to ensure the nature of the stationary points, which have zero imaginary frequencies (see the Supporting Information for details).

## Supporting Information Available

Experimental details and characterization procedures for the compounds prepared and NMR, UV–vis, and time-resolved changes and *k*_obs_ trends for the reactions studied (PDF)

## Accession Codes

CCDC 2110263 and 2110264 contain the supplementary crystallographic data for this paper. These data can be obtained free of charge via www.ccdc.cam.ac.uk/data_request/cif, or by emailing data_request@ccdc.cam.ac.uk, or by contacting The Cambridge Crystallographic Data Centre, 12 Union Road, Cambridge CB2 1EZ, UK; fax: +44 1223 336033.
