# Supplementary material for: Molecular Approach to Alkali-Metal Encapsulation by a Prussian Blue Analogue FeII/CoIII Cube in Aqueous Solution: A Kineticomechanistic Exchange Study
Source: Inorg Chem. 2021 Nov 12;60(23):18407–22. doi: 10.1021/acs.inorgchem.1c03001 (PMC8715505; doi:10.1021/acs.inorgchem.1c03001)
Supplement: Supplementary file 1 — ic1c03001_si_001.pdf [file ic1c03001_si_001.pdf]

Supporting Information

Molecular approach to alkali metal encapsulation by a PrussianBlueAnalogue Fe^{II}/Co^{III} cube in aqueous solution, kinetico-mechanistic exchange study.

Miguel A. Gonzálvez,[a,b] *Paul V. Bernhardt,*[a] *Mercè Font-Bardia,*[c] *Albert Gallen,*[b] *Jesús Jover,*[b,d] *Montserrat Ferrer,*[b,e*] *and Manuel Martínez* [b,e*]

[a] School of Chemistry and Molecular Biosciences, University of Queensland, Brisbane, Queensland 4072, Australia

[b] Secció de Química Inorgànica, Departament de Química Inorgànica i Orgànica. Universitat de Barcelona, Martí i Franquès 1-11, 08028 Barcelona, Spain

[c] Unitat de Difracció de Raigs-X. Centre Científic i Tecnològic de la Universitat de Barcelona. Universitat de Barcelona and Departament de Cristal·lografia, Mineralogia i Dipòsits Minerals. Facultat de Geologia, 08028 Barcelona, Spain

[d] Institut de Química Teòrica i Computacional (IQTC-UB), Universitat de Barcelona, 08028 Barcelona, Spain

[e] Institute of Nanoscience and Nanotechnology (IN2UB), Universitat de Barcelona, 08028 Barcelona, Spain

montse.ferrer@qi.ub.edu, manel.martinez@qi.ub.edu

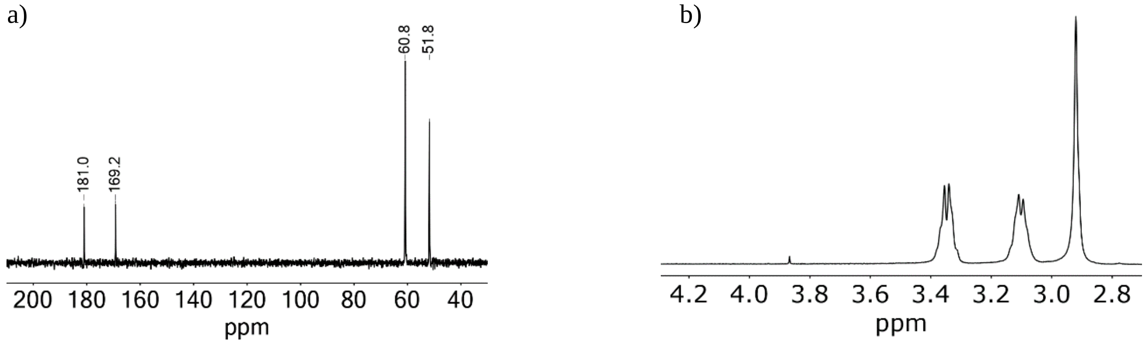

Figure S1.- a) ^{13}C and b) ^{1}H NMR spectra of the void lithium salt of the cubic $[\{Co^{III}(Me_3\text{-}tacn)\}_4\{Fe^{II}(CN)_6\}_4]^{4-}$ structure. The ^{1}H NMR spectrum shows a minor signal at 3.87 ppm due to the presence of residual sodium salt.

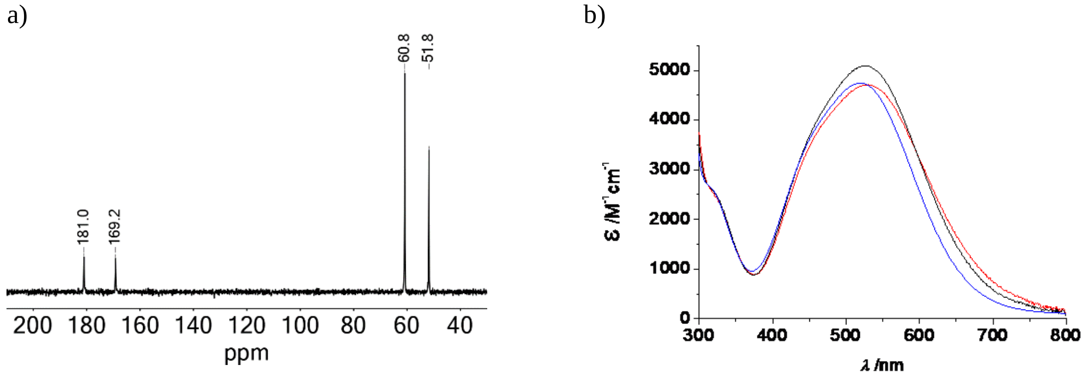

Figure S2.- a) ^{13}C NMR spectra of the rubidium salt of the cubic $[\{Co^{III}(Me_3\text{-}tacn)\}_4\{Fe^{II}(CN)_6\}_4]^{4-}$ structure. b) Electronic spectra of the rubidium (black), sodium (red) and potassium (blue) salts of the $[\{Co^{III}(Me_3\text{-}tacn)\}_4\{Fe^{II}(CN)_6\}_4]^{4-}$ cubic cages in water.

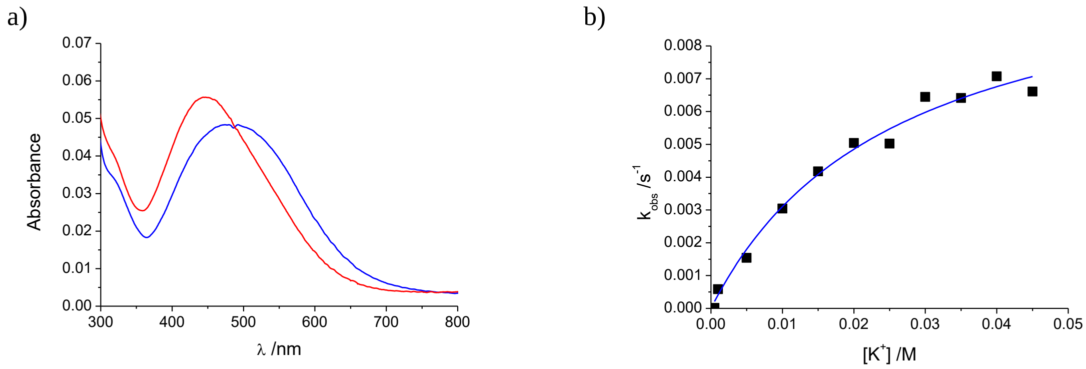

Figure S3.- a) Electronic spectra of the sodium (red) and potassium (blue) salts of the $[\{Co^{III}(Me_3\text{-}tacn)\}_4\{Fe^{II}(CN)_6\}_4]^{4-}$ cubic cages in 0.1 M HCl at a saturated 1.5×10^{-5} M concentration. b) $[K^+]$-dependence at 15 °C in 0.1 M HCl solution of the values of k_{obs} for the exchange from the sodium to the potassium $[\{Co^{III}(Me_3\text{-}tacn)\}_4\{Fe^{II}(CN)_6\}_4]^{4-}$ cubic cages.

a) b)

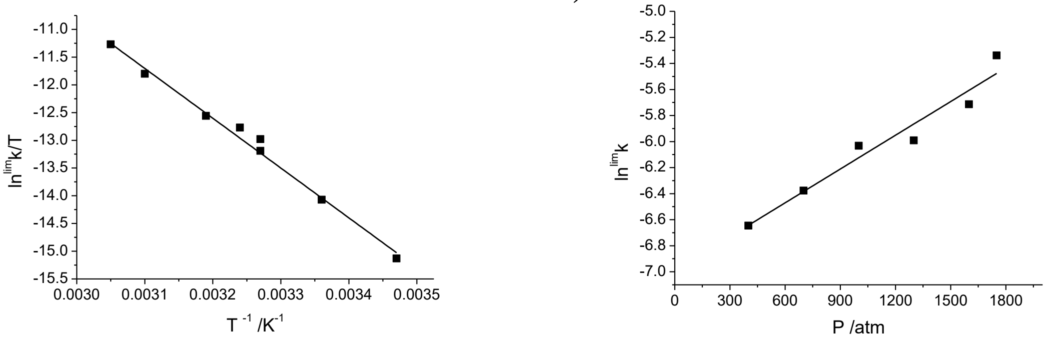

Figure S4.- Eyring , a), and ln*k* *versus* *P*, b), plots for the variations of the values of the limiting value of k_{obs} for the Rb^+ for $\{NaOH_2\}^+$ exchange in the $\{\{Co^{III}(Me_3\text{-tacn})\}_4\{Fe^{II}(CN)_6\}_4\}$ cubic structure.

a) b)

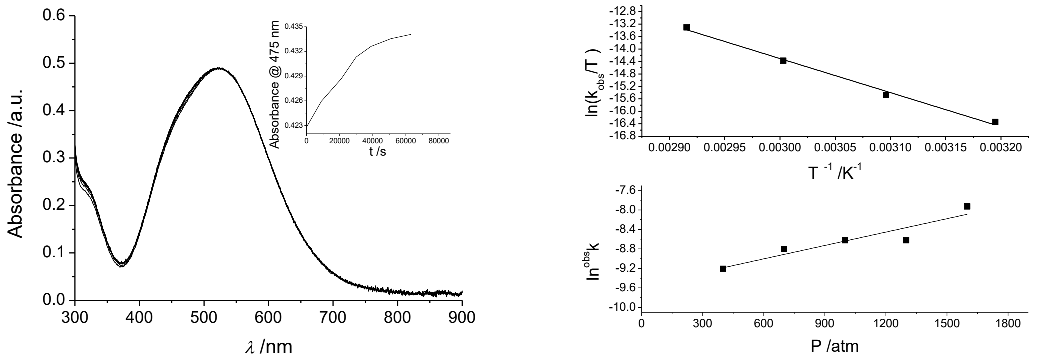

Figure S5.- a) Time-resolved UV-Vis spectral changes observed on solution of a sample of a lithium salt of the $[\{Co^{III}(Me_3\text{-tacn})\}_4\{Fe^{II}(CN)_6\}_4]^{4-}$ cubic structure containing encapsulated $\{LiOH_2\}^+$ units (T = 40 ºC). b) Eyring (top) and ln*k* *versus* *P* (bottom) plots for the variations of the values of the value of k_{obs} for the same experiments.

a) b)

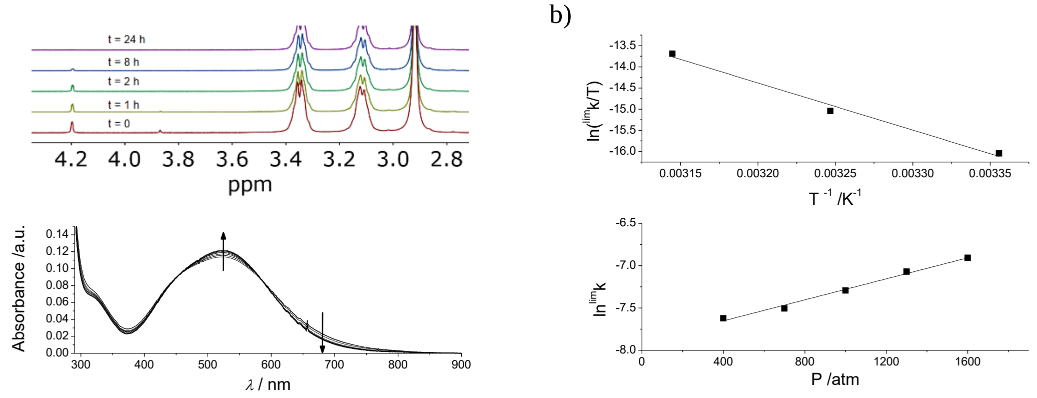

Figure S6.- a) Time-resolved ^1H NMR (top) and UV-Vis (bottom) spectral changes observed on solution of a sample of a lithium salt of the $[\{Co^{III}(Me_3\text{-tacn})\}_4\{Fe^{II}(CN)_6\}_4]^{4-}$ cubic structure containing encapsulated $\{LiOH_2\}^+$ units in 0.10 M KCl. b) Eyring (top) and ln*k* *versus* *P* (bottom) plots for the variations of the limiting value of k_{obs} for this series of experiments.

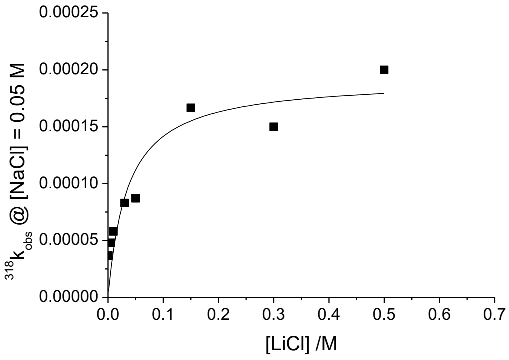

Figure S7.- Effect of the [LiCl] added on the value of the observed rate constants for the confined cation exchange of $\{LiOH_2\}^+$ by $\{NaOH_2\}^+$ at $[\{\{Co^{III}(Me_3\text{-tacn})\}_4\{Fe^{II}(CN)_6\}_4\}] = 5\times10^{-5}$ M and at 0.05 M [NaCl] (45 ºC).

Table S1.- Values of the observed rate constants for the cation exchange processes studied as a function of ion concentration, ionic strength, acidity temperature, and pressure. [$\{Fe^{II}_4Co^{III}_4\}^{4-}$] = (2-10)×$10^{-5}$ M.

Cation exchange	Medium	[entering cation] /M	Ionic strength /M	*T* /ºC	*P* /atm	$10^4\times k_{obs}$ /s^{-1}
$\{NaOH_2\}^+$ to K^+	H_2O	0.15	--	25	1	1.6
		0.00010	1.0 $LiClO_4$	25	1	2.5
		0.0050	1.0 $LiClO_4$	25	1	2.7
		0.010	1.0 $LiClO_4$	25	1	2.6
		0.020	1.0 $LiClO_4$	25	1	2.8
		0.030	1.0 $LiClO_4$	25	1	2.8
		0.040	1.0 $LiClO_4$	25	1	2.8
		0.050	1.0 $LiClO_4$	25	1	2.9
		0.00070	3.0 $LiClO_4$	25	1	3.3
		0.0045	3.0 $LiClO_4$	25	1	3.1
		0.015	3.0 $LiClO_4$	25	1	2.9
		0.010	--	35	1	5.7
		0.0050	--	35	1	5.7
		0.0010	--	35	1	5.6
		0.00050	--	35	1	5.5
		0.025	0.10 NaCl	35	1	5.7
		0.020	0.10 NaCl	35	1	5.5
		0.015	0.10 NaCl	35	1	5.4
		0.010	0.10 NaCl	35	1	5.3
		0.0050	0.10 NaCl	35	1	4.9
		0.0010	0.10 NaCl	35	1	3.1
		0.00050	0.10 NaCl	35	1	5.7
		0.025	0.50 NaCl	35	1	5.0
		0.020	0.50 NaCl	35	1	4.8
		0.015	0.50 NaCl	35	1	4.5
		0.010	0.50 NaCl	35	1	3.9
		0.0050	0.50 NaCl	35	1	3.0
		0.0010	0.50 NaCl	35	1	1.5
		0.04	1.0 $NaClO_4$	35	1	5.3
		0.035	1.0 $NaClO_4$	35	1	5.4
		0.03	1.0 $NaClO_4$	35	1	4.9
		0.025	1.0 $NaClO_4$	35	1	4.6
		0.02	1.0 $NaClO_4$	35	1	4.0
		0.015	1.0 $NaClO_4$	35	1	3.5
		0.01	1.0 $NaClO_4$	35	1	2.7
		0.005	1.0 $NaClO_4$	35	1	1.8
		0.0010	1.0 $NaClO_4$	35	1	0.82
		0.0005	1.0 $NaClO_4$	35	1	0.64
		0.15	--	35	400	5.5
		0.15	--	35	700	9.5
		0.15	--	35	1000	7.8
		0.15	--	35	1300	13
		0.15	--	35	1600	15
		0.15	--	45	1	18
	0.1 M HCl	0.10	--	9.5	1	52
		0.10	--	15	1	100
		0.10	--	25	1	180
		0.045	--	25	1	66
		0.040	--	25	1	71
		0.035	--	25	1	64
		0.030	--	25	1	65
		0.025	--	25	1	50
		0.020	--	25	1	50
		0.015	--	25	1	42
		0.010	--	25	1	30

		0.0050	--	25	1	15
		0.0010	--	25	1	5.8
		0.10	--	35	1	380
$\{NaOH_2\}^+$ to Rb^+	H_2O	0.20	--	15	1	0.77
		0.10	--	25	1	1.9
		0.20	--	25	1	2.5
		0.30	--	25	1	2.2
		0.40	--	25	1	2.3
		0.50	--	25	1	2.3
		0.050	0.10 NaCl	25	1	0.12
		0.10	0.050 NaCl	25	1	0.12
		0.10	0.10 NaCl	25	1	0.097
		0.10	0.10 NaCl	25	1	0.068
		0.0010	0.50 NaCl	25	1	0.097
		0.0050	0.50 NaCl	25	1	0.092
		0.010	0.50 NaCl	25	1	0.084
		0.020	0.50 NaCl	25	1	0.098
		0.030	0.50 NaCl	25	1	0.090
		0.040	0.50 NaCl	25	1	0.10
		0.050	0.50 NaCl	25	1	0.46
		0.10	0.60 NaCl	25	1	0.21
		0.050	---	33	1	8.0
		0.20	--	33	1	7.1
		0.15	--	33	1	6.8
		0.15	0.010 NaCl	33	1	1.5
		0.15	0.030 NaCl	33	1	0.71
		0.15	0.050 NaCl	33	1	0.67
		0.15	0.075 NaCl	33	1	0.55
		0.020	--	36	1	8.9
		0.25	--	39	400	13
		0.25	--	39	700	17
		0.25	--	39	1000	24
		0.25	--	39	1300	25
		0.25	--	39	1600	33
		0.25	--	39	1750	48
		0.040	--	40	1	11
		0.10	--	50	1	24
		0.20	--	55	1	42
$\{LiOH_2\}^+$ to void	H_2O	--	--	40	1	0.25
		--	--	50	1	0.61
		--	--	55	400	1.0
		--	--	55	700	1.5
		--	--	55	1000	1.8
		--	--	55	1300	1.8
		--	--	55	1600	2.8
		--	--	60	1	1.9
		--	--	70	1	5.4
$\{LiOH_2\}^+$ to $\{NaOH_2\}^+$	H_2O	0.10	0.050 $LiClO_4$	35	1	1.2
		0.25	0.030 $LiClO_4$	35	1	0.42
		0.25	0.040 LiCl	35	1	0.72
		0.050	0.0010 LiCl	45	1	0.36
		0.050	0.0050 LiCl	45	1	0.48
		0.050	0.010 LiCl	45	1	0.58
		0.050	0.030 LiCl	45	1	0.83
		0.050	0.050 LiCl	45	1	0.87
		0.050	0.15 LiCl	45	1	1.7
		0.050	0.30 LiCl	45	1	1.5
		0.050	0.50 LiCl	45	1	1.8
		0.10	0.050 LiCl	45	1	1.2
		0.10	0.050 LiCl	46	400	1.1
		0.10	0.050 LiCl	46	700	1.0
		0.10	0.050 LiCl	46	1000	1.0
		0.10	0.050 LiCl	46	1300	0.98

		0.10	0.050 LiCl	46	1600	0.92
		0.10	0.050 LiCl	46	1800	0.95
		0.050	0.050 LiCl	55	1	5.9
		0.10	0.050 LiCl	55	1	6.0
$\{LiOH_2\}^+$ to K^+	H_2O	0.0050	--	25	1	0.35
		0.015	--	25	1	0.32
		0.10	--	25	1	0.32
		0.10	--	35	1	0.90
		0.10	0.05 LiCl	35	1	0.92
		0.10	0.20 LiCl	35	1	0.78
		0.10	0.50 LiCl	35	1	0.88
		0.10	1.0 LiCl	35	1	0.77
		0.0050	--	45	1	3.8
		0.015	--	45	1	3.9
		0.10	--	45	1	3.6
		0.10	--	46	400	4.9
		0.10	--	46	700	5.5
		0.10	--	46	1000	6.8
		0.10	--	46	1300	8.5
		0.10	--	46	1600	10
$\{LiOH_2\}^+$ to Rb^+	H_2O	0.10	--	35	1	1.4
		0.010	--	45	1	3.5
		0.010	0.050 LiCl	45	1	3.5
		0.10	--	45	1	3.1
		0.10	--	46	400	5.9
		0.10	--	46	700	8.4
		0.10	--	46	1000	8.7
		0.10	--	46	1300	12
		0.10	--	46	1600	14
		0.10	--	55	1	8.8

Table S2.- Crystal data and structure refinement for $Li_8\{\{LiOH_2\}\subset[\{Co^{III}(Me_3\text{-}tacn)\}_4\{Fe^{II}(CN)_6\}_4]\}\cdot 5ClO_4\cdot 12H_2O$.

Identification code	iapzb119_0ma_a	
Empirical formula	C60 H110 Cl5 Co4 Fe4 Li9 N36 O33	
Formula weight	2562.66	
Temperature	100(2) K	
Wavelength	0.71073 Å	
Crystal system	Cubic	
Space group	$F\bar{4}3m$	
Unit cell dimensions	a = 23.4538(7) Å	α= 90°.
	b = 23.4538(7) Å	β= 90°.
	c = 23.4538(7) Å	γ = 90°.
Volume	12901.5(12) $Å^3$	
Z	4	
Density (calculated)	1.319 Mg/m^3	
Absorption coefficient	1.115 mm^{-1}	
F(000)	5240	
Crystal size	0.180 x 0.130 x 0.130 mm^3	
Theta range for data collection	2.456 to 24.966°.	
Index ranges	-33<=h<=33, -29<=k<=33, -32<=l<=29	
Reflections collected	1167	
Independent reflections	1167 [R(int) = ?]	
Completeness to theta = 24.966°	99.5 %	
Refinement method	Full-matrix least-squares on F^2	
Data / restraints / parameters	1167 / 7 / 69	
Goodness-of-fit on F^2	1.127	
Final R indices [I>2sigma(I)]	R1 = 0.0894, wR2 = 0.2678	
R indices (all data)	R1 = 0.1001, wR2 = 0.2806	
Absolute structure parameter	0.068(19)	
Extinction coefficient	n/a	
Largest diff. peak and hole	0.783 and -0.764 e.$Å^{-3}$	
CDCC reference	2110263	

Table S3.- Crystal data and structure refinement for $Na_3\{\{NaOH_2\}\subset[\{Co^{III}(Me_3$-tacn$)\}_4\{Fe^{II}(CN)_6\}_4]\}\cdot 22H_2O$.

Identification code	iapzb24_b_sq_sa	
Empirical formula	C60 H130 Co4 Fe4 N36 Na4 O23	
Formula weight	2275.07	
Temperature	100(2) K	
Wavelength	0.71073 Å	
Crystal system	Tetragonal	
Space group	P -4 21 c	
Unit cell dimensions	a = 17.1159(9) Å	α= 90°.
	b = 17.1159(9) Å	β= 90°.
	c = 18.6353(11) Å	γ = 90°.
Volume	5459.3(7) $Å^3$	
Z	2	
Density (calculated)	1.384 Mg/m^3	
Absorption coefficient	1.198 mm^{-1}	
F(000)	2364	
Crystal size	0.460 x 0.270 x 0.180 mm^3	
Theta range for data collection	2.380 to 25.000°.	
Index ranges	-14<=h<=14, -20<=k<=20, -22<=l<=22	
Reflections collected	4807	
Independent reflections	4807 [R(int) = 0.0694]	
Completeness to theta = 25.000°	99.8 %	
Refinement method	Full-matrix least-squares on F^2	
Data / restraints / parameters	4807 / 12 / 227	
Goodness-of-fit on F^2	1.099	
Final R indices [I>2sigma(I)]	R1 = 0.0639, wR2 = 0.1633	
R indices (all data)	R1 = 0.0676, wR2 = 0.1653	
Absolute structure parameter	0.16(5)	
Extinction coefficient	n/a	
Largest diff. peak and hole	0.908 and -0.618 e.$Å^{-3}$	
CDCC reference	2110264	

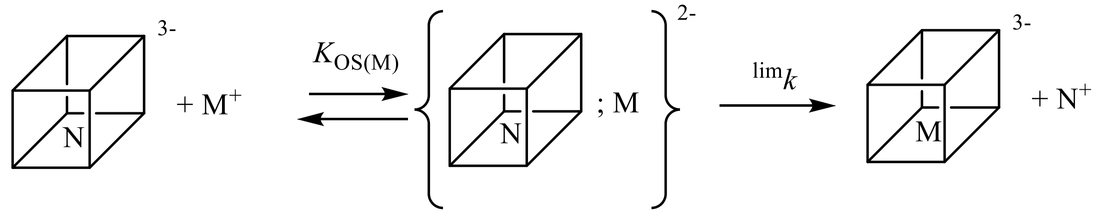

A {**A**;M} **B**

$$d[\mathbf{A}]_{mesurable}/dt = v = {}^{lim}k\ [\{\mathbf{A};M\}] = {}^{lim}k\ K_{OS(M)}\ [\mathbf{A}]\ [M]$$

As $[\mathbf{A}]_{measurable} = [\mathbf{A}] + [\{\mathbf{A};M\}]$

$$k_{obs} = \frac{{}^{lim}k\ K_{OS(M)}\ [\mathbf{A}]\ [M]}{1 + K_{OS(M)}\ [M]}$$

Scheme S1.- Applicable Eigen-Wilkins mechanism for the reactions studied.

Experimental

Physical Methods

DOSY NMR measurements were performed on a Bruker 400 MHz NMR spectrometer equipped with a 5 mm CPPBBO BB-1H/19F/D Z-GRD probe with a maximum strength of 5.5 G/mm at 298 K. The 90 pulse length was determined for sample. A standard LEDBPGP2S sequence was used with sinusoidal pulse field gradients and two spoiler gradients. A recovery delay of 150 μs and an LED delay of 5 ms were employed to reduce eddy current effects. The gradient strength was calibrated by using the self-diffusion coefficient of residual HOD in D_2O ($1.9x10^{-9}$ m^2 s^{-1}). The gradient strength was increased from 2 – 95% in sixteen equally spaced steps with eight scans per increment. Values of δ (gradient pulse length) and Δ (diffusion time) were selected to give an intensity of about 5% of the initial intensity at 95% gradient strength. MNova software was used to process data.

The general kinetic procedures have been already described,[1-2] and the observed rate constants were derived from absorbance versus time traces at the wavelengths where a maximum increase and/or decrease of absorbance were observed. The calculation of the observed rate constants from the absorbance *versus* time monitoring of reactions, studied under first order concentration conditions (cubic cages in defect), were carried out using the SPECFIT or ReactLab software packages.[3-4] All post-run fittings were carried out by the standard available commercial programs. Table S1 collects all the values obtained for the experiments run.

X-Ray Structure Analysis

For the lithium derivative of the cubic structure, the frames were integrated with the Bruker SAINT software package using a narrow-frame algorithm.[5] The integration of the data using a cubic unit cell yielded a total of 1167 reflections to a maximum θ angle of 24.97° (0.84 Å resolution), of which 1167 were independent (average redundancy 1.000, completeness = 99.5%, R_{sig} = 2.09%) and 949 (81.32%) were greater than $2\sigma(F^2)$. The final cell constants of a = 23.4538(7) Å, b = 23.4538(7) Å, c = 23.4538(7) Å, volume = 12901.5(12) $Å^3$, are based upon the refinement of the XYZ-centroids of reflections above 20 σ(I). Data were corrected for absorption effects using the Multi-Scan method (SADABS).[6] The structure was solved and refined using the Bruker SHELXTL Software Package,[7] using the cubic space group $F\bar{4}3m$, with Z = 4 for the formula unit, $C_{60}H_{110}Cl_5Co_4Fe_4Li_9N_{36}O_{33}$. The final anisotropic full-matrix least-squares refinement on F^2 with 74 variables converged at R1 = 8.03%, for the observed data and wR2 = 24.72% for all data. The goodness-of-fit was 1.087. The largest peak in the final difference electron density synthesis was 0.792 $e^-/Å^3$ and the largest hole was -0.392 $e^-/Å^3$ with an RMS deviation of 0.106 $e^-/Å^3$. On the basis of the final model, the calculated density was 1.319 g/cm^3 and F(000), 5240 e^-.

Table S2 collects the relevant crystallographic data. As a whole the cube is well defined, the Me_3-tacn ligand is conformationally disordered. The bridging CN^- ligands are well defined but the terminal CN^- ligands have quite high thermal parameters and were allowed to split into two components and were refined with complementary occupancies. The number of Li^+ ions inside the cage cannot be determined

with certainty by XRD. Nevertheless, the apparent electron density between the central O atom and the Co atom occupying a 3*m* site can be associated to a Li^+ ion in this position with a 35 % occupancy, which gives a reasonable thermal parameter with rather short Li-O bond length, not unrealistic given the constraints within the cube. More Li^+ could be fitted inside the cubic architecture, but the ^1H and ^7Li NMR data do not agree with such an approach.

As for the sodium derivative of the cubic structure, the frames were integrated with the Bruker SAINT software package using a narrow-frame algorithm.[5] The integration of the data using a tetragonal unit cell yielded a total of 4807 reflections to a maximum θ angle of 25.00 ° (0.84 Å resolution), of which 4807 were independent (average redundancy 1.000, completeness = 99.8 %, R_{int} = 6.94 %, R_{sig} = 3.95 %) and 4469 (92.97 %) were greater than $2\sigma(F^2)$. The final cell constants of a = 17.1159(9) Å, b = 17.1159(9) Å, c = 18.6353(11) Å, volume = 5459.3(7) $Å^3$, are based upon the refinement of the XYZ-centroids of reflections above 20 σ(I). Data were corrected for absorption effects using the Multi-Scan method (SADABS).[6] The structure was solved and refined using the Bruker SHELXTL Software Package,[7] using the space group $P\,\bar{4}\,2_1c$, with Z = 2 for the formula unit, $C_{60}H_{130}Co_4Fe_4N_{36}Na_4O_{23}$. The final anisotropic full-matrix least-squares refinement on F^2 with 227 variables converged at R1 = 6.39 %, for the observed data and wR2 = 16.53 % for all data. The goodness-of-fit was 1.099. The largest peak in the final difference electron density synthesis was 0.908 $e^-/Å^3$ and the largest hole was -0.618 $e^-/Å^3$ with an RMS deviation of 0.106 $e^-/Å^3$. On the basis of the final model, the calculated density was 1.384 g/cm^3 and F(000), 2364 e^-.

Table S3 collects the relevant crystallographic data. The structure encapsulates a single Na^+ ion and a water molecule both at 25 % occupancy; the Na^+ ion adopts a pseudo square planar geometry perched above one face of the cube The Na-O distance is quite short (2.09(2) Å) compared with typical Na-O distances but not unrealistic given the constraints within the cube.

Characterisation of compounds

<u>Lithium salt of $[\{Co^{III}(Me_3\text{-tacn})\}_4\{Fe^{II}(CN)_6\}_4]^{4-}$, from Sephadex G-25 chromatography:</u>

ICP-OES: Ratio Fe:Co 1.02±0.01

^1H NMR (400 MHz, D_2O, 298 K) (δ/ppm): 2.92 (s br, 36 H, CH_3), 3.04-3.16 (m, 24H, CH_2 *anti*), 3.29-3.41 (m, 24H, CH_2 *syn*).

^{13}C NMR (100.6 MHz, D_2O, 298 K) (δ/ppm): 51.8 (s, CH_3), 60.9 (s, CH_2), 169.2 (s, terminal C≡N), 181.0 (s, bridging C≡N).

^7Li NMR (194.2 MHz, D_2O, 298 K) (δ/ppm): 0 (Li^+_{aq}).

UV-Vis (H_2O): {nm (ε /M^{-1} cm^{-1})}: 320 (2100), 467 (sh, 3400), 523 (4300).

IR (ATR) (cm^{-1}): 2161 m, 2134 s, 2058 vs, ν(C≡N).

Electrochemistry: $Fe^{III/II}$ potentials (mV *vs.* NHE): 825, 975, 1140, 1323.

<u>Lithium salt of $[\{Co^{III}(Me_3\text{-tacn})\}_4\{Fe^{II}(CN)_6\}_4]^{4-}$, from elution of the sodium salt with $LiClO_4$ of Sephadex DEAE A-25 chromatography:</u>

ICP-OES: Ratio Fe:Co 0.98±0.01

^{1}H NMR (400 MHz, D_2O, 298 K) (δ/ppm): 2.92 (s br, 36 H, CH_3), 3.04-3.16 (m, 24H, CH_2 *anti*), 3.29-3.41 (m, 24H, CH_2 *syn*), 4.21 (q, $^{2}J_{HLi}$ = 1.1 Hz, $H_2O_{encapsulated}$).

^{13}C NMR (100.6 MHz, D_2O, 298 K) (δ/ppm): 51.8 (s, CH_3), 60.9 (s, CH_2), 169.3 (s, terminal C≡N), 182.5 (s, bridging C≡N).

^{7}Li NMR (194.2 MHz, D_2O, 298 K) (δ/ppm): 0 (Li^{+}_{aq}), 0.09 (t br, $^{2}J_{LiH}$ = 1.0 Hz, $Li^{+}_{encapsulated}$).

UV-Vis (H_2O): {nm (ε /M^{-1} cm^{-1})}: 320 (2100), 470 (sh, 3500), 524 (4050).

IR (ATR) (cm^{-1}): 2158 m, 2140 s, 2061 vs, ν(C≡N).

Electrochemistry: Fe oxidation peaks (mV *vs.* NHE): 772, 895; Fe reduction peaks (mV *vs.* NHE): 871, 715, 556, 377.

<u>Sodium salt of $[\{Co^{III}(Me_3\text{-tacn})\}_4\{Fe^{II}(CN)_6\}_4]^{4-}$:</u>

ICP-OES: Ratio Fe-Co 1.01±0.01

^{1}H NMR (500 MHz, D_2O, 298 K) (δ/ppm): 2.92 (s br, 36 H, CH_3), 3.04-3.16 (m, 24H, CH_2 *anti*), 3.29-3.41 (m, 24H, CH_2 *syn*), 3.87 (s, 2H, $H_2O_{encapsulated}$).

^{13}C NMR (100.6 MHz, D_2O, 298 K) (δ/ppm): 51.8 (s, CH_3), 60.9 (s, CH_2), 169.4 (s, terminal C≡N), 182.0 (s, bridging C≡N).

^{23}Na NMR (132.3 MHz, D_2O, 298 K) (δ/ppm): 0 (Na^{+}_{aq}), 5 (s br, $Na^{+}_{encapsulated}$).

UV-Vis (H_2O): {nm (ε /M^{-1} cm^{-1})}: 324 (2740), 457 (sh, 3820), 525 (4710).

IR (ATR) (cm^{-1}): 2148 m, 2123 s, 2055 vs, ν(C≡N).

Electrochemistry: Fe oxidation peaks (mV *vs.* NHE): 889, 1156; Fe reduction peaks (mV *vs.* NHE): 1089, 856, 684, 457.

<u>Potassium salt of $[\{Co^{III}(Me_3\text{-tacn})\}_4\{Fe^{II}(CN)_6\}_4]^{4-}$:</u>

ICP-OES: Ratio Fe-Co 1.03±0.01

^{1}H NMR (400 MHz, D_2O, 298 K): (δ/ppm) 2.92 (s br, 36H, CH_3), 3.07-3.19 (m, 24H, CH_2 *anti*), 3.30-3.42 (m, 24H, CH_2 *syn*).

^{13}C NMR (100.6 MHz, D_2O, 298 K) (δ/ppm}: 52.0 (s, CH_3), 61.0 (s, CH_2), 169.1 (s, terminal C≡N), 181.00 (s, bridging C≡N).

UV-Vis (H_2O) {nm (ε /M^{-1} cm^{-1})}: 317 (2600), 455 (sh, 3950), 518 (4740).

IR (ATR) (cm^{-1}): 2151 m, 2125 s, 2063 vs, ν(C≡N).

Electrochemistry: $Fe^{III/II}$ potentials (mV *vs.* NHE): 821, 970, 1050, 1310.

Computational details

The DFT optimized structures can be freely accessed through the ioChem-BD repository: DOI: https://doi.org/10.19061/iochem-bd-1-212.

References

1. Aullón, G.; Crespo, M.; Jover, J.; Martínez, M., Chapter Five - Diarylplatinum(II) Scaffolds for Kinetic and Mechanistic Studies on the Formation of Platinacycles via an Oxidative Addition/Reductive Elimination/Oxidative Addition Sequence. In *Advances in Inorganic Chemistry; Inorganic Reaction Mechanisms*, Volume 70 ed.; Eldik, R. v.; Hubbard, C., Eds. Academic Press: 2017; pp 195-242.
2. Juribasi, M.; Budimir, A.; Kazazic, S.; Curic, M., Dicyclopalladated Complexes of Asymmetrically Substituted Azobenzenes: Synthesis, Kinetics and Mechanisms. *Inorganic Chemistry* **2013,** *52*, 12749-12757.
3. Maeder, M.; King, P. *ReactLab*, Jplus Consulting Pty Ltd: East Fremantle, WA. Australia, 2009.
4. Binstead, R. A.; Zuberbuhler, A. D.; Jung, B. *SPECFIT32*, 3.0.34; Spectrum Software Associates: Marlborough, MA, USA, 2005.
5. *SAINT* [7.68A]; Bruker AXS Inc. : 2009.
6. Sheldrick, G. M. *SADABS*, 2008/1; Bruker AXS Inc.: 2008.
7. Sheldrick, G. M., SHELXL. *Acta Crystallographica Section A* **2008,** *A64*, 112-122.
